# Transcription factors MhDREB2A/MhZAT10 play a role in drought and cold stress response crosstalk in apple

**DOI:** 10.1093/plphys/kiad147

**Published:** 2023-03-06

**Authors:** Xing-Liang Li, Dong Meng, Min-Ji Li, Jia Zhou, Yu-Zhang Yang, Bei-Bei Zhou, Qin-Ping Wei, Jun-Ke Zhang

**Affiliations:** Key Laboratory of Biology and Genetic Improvement of Horticultural Crops (North China), Institute of Forestry and Pomology, Beijing Academy of Agriculture and Forestry Sciences, Beijing 100093, People’s Republic of China; Key Laboratory of Biology and Genetic Improvement of Horticultural Crops (North China), Institute of Forestry and Pomology, Beijing Academy of Agriculture and Forestry Sciences, Beijing 100093, People’s Republic of China; Key Laboratory of Biology and Genetic Improvement of Horticultural Crops (North China), Institute of Forestry and Pomology, Beijing Academy of Agriculture and Forestry Sciences, Beijing 100093, People’s Republic of China; Key Laboratory of Biology and Genetic Improvement of Horticultural Crops (North China), Institute of Forestry and Pomology, Beijing Academy of Agriculture and Forestry Sciences, Beijing 100093, People’s Republic of China; Key Laboratory of Biology and Genetic Improvement of Horticultural Crops (North China), Institute of Forestry and Pomology, Beijing Academy of Agriculture and Forestry Sciences, Beijing 100093, People’s Republic of China; Key Laboratory of Biology and Genetic Improvement of Horticultural Crops (North China), Institute of Forestry and Pomology, Beijing Academy of Agriculture and Forestry Sciences, Beijing 100093, People’s Republic of China; Key Laboratory of Biology and Genetic Improvement of Horticultural Crops (North China), Institute of Forestry and Pomology, Beijing Academy of Agriculture and Forestry Sciences, Beijing 100093, People’s Republic of China; Key Laboratory of Biology and Genetic Improvement of Horticultural Crops (North China), Institute of Forestry and Pomology, Beijing Academy of Agriculture and Forestry Sciences, Beijing 100093, People’s Republic of China

## Abstract

Drought and cold stresses seriously affect tree growth and fruit yield during apple (*Malus domestica*) production, with combined stress causing injury such as shoot shriveling. However, the molecular mechanism underlying crosstalk between responses to drought and cold stress remains to be clarified. In this study, we characterized the zinc finger transcription factor ZINC FINGER OF ARABIDOPSIS THALIANA 10 (ZAT10) through comparative analysis of shoot-shriveling tolerance between tolerant and sensitive apple rootstocks. MhZAT10 responded to both drought and cold stresses. Heterologous expression of *MhZAT10* in the sensitive rootstock ‘G935' from domesticated apple (*Malus domestica*) promoted shoot-shriveling tolerance, while silencing of *MhZAT10* expression in the tolerant rootstock ‘SH6' of *Malus honanensis* reduced stress tolerance. We determined that the apple transcription factor DEHYDRATION RESPONSE ELEMENT-BINDING PROTEIN 2A (DREB2A) is a direct regulator activating the expression of *MhZAT10* in response to drought stress. Apple plants overexpressing both *MhDREB2A* and *MhZAT10* genes exhibited enhanced tolerance to drought and cold stress, while plants overexpressing *MhDREB2A* but with silenced expression of *MhZAT10* showed reduced tolerance, suggesting a critical role of MhDREB2A-MhZAT10 in the crosstalk between drought and cold stress responses. We further identified drought-tolerant *MhWRKY31* and cold-tolerant *MhMYB88* and *MhMYB124* as downstream regulatory target genes of MhZAT10. Our findings reveal a MhDREB2A-MhZAT10 module involved in crosstalk between drought and cold stress responses, which may have applications in apple rootstock breeding programs aimed at developing shoot-shriveling tolerance.

## Teaser

The transcription factor MhDREB2A activates *MhZAT10* expression, and they coordinately regulate drought and cold stress by integrating multiple pathways in apple rootstock to improve shoot-shriveling tolerance.

## Introduction

Climate change typically causes comprehensive harm to plant growth. Abiotic stresses such as drought, cold, and high-salt and biological stresses such as pests and diseases often affect plant growth and development simultaneously, resulting in reduced yield and fruit quality. In cold regions, such as northern China, the frozen soil layer is thick in winter, enveloping the whole root systems of fruit trees. When the aboveground temperature rises in early spring, the soil has not thawed completely and roots remain dormant underground; at the same time, transpiration increases in the branches with insufficient water supply. Thus, fruit trees suffer a drought and cold combined stress, which often leads to shoot shriveling (Li et al. 2010; Liang et al. 2017). Shoot shriveling caused by the superimposition of drought and cold stress occurs especially in young 1- to 3-year-old fruit trees of apple (*Malus domestica*), pear (*Pyrus communis*), apricot (*Prunus armeniaca*), peach (*Prunus persica*), and other fruits, resulting in tree shape disorder, incomplete crowns, and even death of all aboveground parts. This drought and cold combined stress is one of the main factors restricting the development of young orchards.

Drought and cold are two of the most common abiotic stresses limiting the geographical distribution and growth and development of plant species and causing devastating effects on fruit yield. Both drought and cold stresses inhibit plant cell membrane fluidity, cause osmotic stress, induce reactive oxygen species (ROS), disrupt protein enzyme catalysis and photosynthesis, and ultimately lead to growth inhibition (Considine and Foyer 2021). However, a variety of physiological and biochemical reactions at the plant cell and whole organism levels create a complex response mechanism. Adaptation of plants to environmental stress depends on the activation of a cascade of molecular networks involving stress perception, signal transduction, and the expression of specific stress-related genes and metabolites (Zhang et al. 2020). Previous studies have revealed details of the signal transduction pathways activated by drought or cold individually, including abscisic acid (ABA)-dependent and ABA-independent pathways activated by drought stress (Haak et al. 2017; Soma et al. 2021) and the INDUCER OF CBF EXPRESSION (ICE)-CBF-COR (COLD-REGULATED) and C-REPEAT/DRE BINDING FACTOR (CBF)-independent signal pathways responding to cold stress (Gong et al. 2020; Hwarari et al. 2022).

Crosstalk among gene regulatory networks involved in drought and cold stress is being increasingly revealed. Cold stress induces the expression of about 40% of drought-responsive rice genes (Gao et al. 2007). Overlap between the expression profiles of stress-responsive genes induced by drought and cold stress has been observed in apple (Li et al. 2017a; Li et al. 2019a), tangerine (*Citrus)* (de Oliveira et al. 2011), grape (*Vitis vinifera*) (Zandkarimi et al. 2015), Arabidopsis (*Arabidopsis thaliana*) (Seki et al. 2002), and other plant species. In addition, overexpression of genes encoding the transcription factor (TF) MYB121 in *Malus* (Cao et al. 2013), the antioxidant enzyme manganese superoxide dismutase (Mn-SOD) in *Populus davidiana* (*Populus*) (Yu et al. 2010), the trihelix TF family member cold-induced GT (poly [dG-dT] factor) (CIGT) from the wild tomato species *Solanum habrochaites* (Yu et al. 2018), and others improves plant tolerance to both drought and cold stresses. Expression of stress response factors is usually related to DNA regulatory elements, and it has been demonstrated that C repeat/dehydration response element (CRT/DRE) plays an important role in responses to drought, cold, and high-salt stress through an ABA-independent pathway (Gong et al. 2020). Multiple types of TFs bind to CRT/DRE, among which DEHYDRATION RESPONSE ELEMENT-BINDING PROTEIN 1 (DREB1)/CBF and DREB2, associated with gene expression in response to cold and drought, have been studied intensely (Chinnusamy et al. 2007; Liao et al. 2017; Wei et al. 2022). Plants overexpressing *AtDREB1a/CBF3* from Arabidopsis, *OsDREB1A* from rice (*Oryza sativa*), and *MbDREB1* from dwarf apple (*Malus baccata*) display improved tolerance to both cold and drought (Ito et al. 2006; Yang et al. 2011; Lee et al. 2021). DREB2 TFs, mainly DREB2A and DREB2B, have been widely employed for the improvement of drought and high-salt tolerance (Sakuma et al. 2006; Wang et al. 2017a; Meena et al. 2022). These results highlight the integration and complexity of plant responses to drought and cold stresses.

The TF SALT TOLERANCE ZINC FINGER/ZINC FINGER OF ARABIDOPSIS THALIANA 10 (STZ/ZAT10) belonging to the cysteine-2/histidine-2 (C2H2)-type zinc finger protein family was first identified in Arabidopsis; *ZAT10* transcripts are found in most tissues, including leaves, stems, roots, and flowers (Ciftci-Yilmaz and Mittler 2008; Nguyen et al. 2016). Interestingly, expression of *ZAT10* is activated by various stresses including drought, cold, salt, ultraviolet-B (UV-B) light, oxidative stress, and high light, and transgenic Arabidopsis, rice, wheat (*Triticum aestivum*), and poplar plants overexpressing *ZAT10* show enhanced tolerance to most of these abiotic stresses (Rossel et al. 2008; Xiao et al. 2009; Park et al. 2015; He et al. 2019). *ZAT10* was initially identified as a cold tolerance gene belonging to the CBF-independent signaling pathway, downstream of mitogen-activated protein kinases (MAPKs) and upstream of ROS-scavenging enzymes including ascorbate peroxidases (APXs) and catalases (CATs), suggesting that *ZAT10* is a rare multiabiotic stress tolerance gene (Gong et al. 2020). However, despite *ZAT10* having been widely identified, especially in cold tolerance, the TFs directly activating *ZAT10* expression and the target genes of TF ZAT10 remain to be clarified in the drought and cold stress response pathways.

Apple plants are perennials that frequently suffer shoot shriveling, especially in arid and cold areas. Tolerant rootstocks have been adopted for apple cultivation to alleviate this problem. The apple rootstock ‘SH6' (*Malus honanensis*) was bred in northern China during the last century and is highly tolerant to drought and cold stress. SH6 is commonly used as a rootstock conferring tolerance to shoot shriveling. In a previous study, we cloned the *MhZAT10* gene from SH6 plants and identified MhZAT10 as a cold response factor; expression of *MhZAT10* was highly associated with that of *MhDREB2A* (Li et al. 2018), which has long been established as a key drought resistance gene in many plants (Gujjar et al. 2014; Wang et al. 2017b). Moreover, five out of nine antioxidant enzyme genes responding to both drought and cold stresses were predicted to be potential ZAT10 TF target genes in our previous study (Li et al. 2017b), strongly indicating that MhZAT10 could play a crosstalk role in responding to and regulating drought and cold stresses in apple.

In this study, through comparative analysis of shoot-shriveling tolerance in tolerant and sensitive apple rootstocks, we further clarified the critical role of MhZAT10 in the crosstalk between drought and cold stress responses and elucidated its regulatory pathways, including upstream TFs and downstream target genes. Our results show that MhZAT10 integrates with *MhDREB2A* responses to drought and cold stress, playing an active regulatory role in the crosstalk between drought and cold responses and improving plant shoot-shriveling tolerance through a multitarget gene regulatory network. The MhDREB2A*-MhZAT10* pathway therefore shows potential for the genetic improvement of drought and cold tolerance of apple rootstocks.

## Results

### Drought and cold combined stress causes more serious damage than single stress

**Figure 1.**
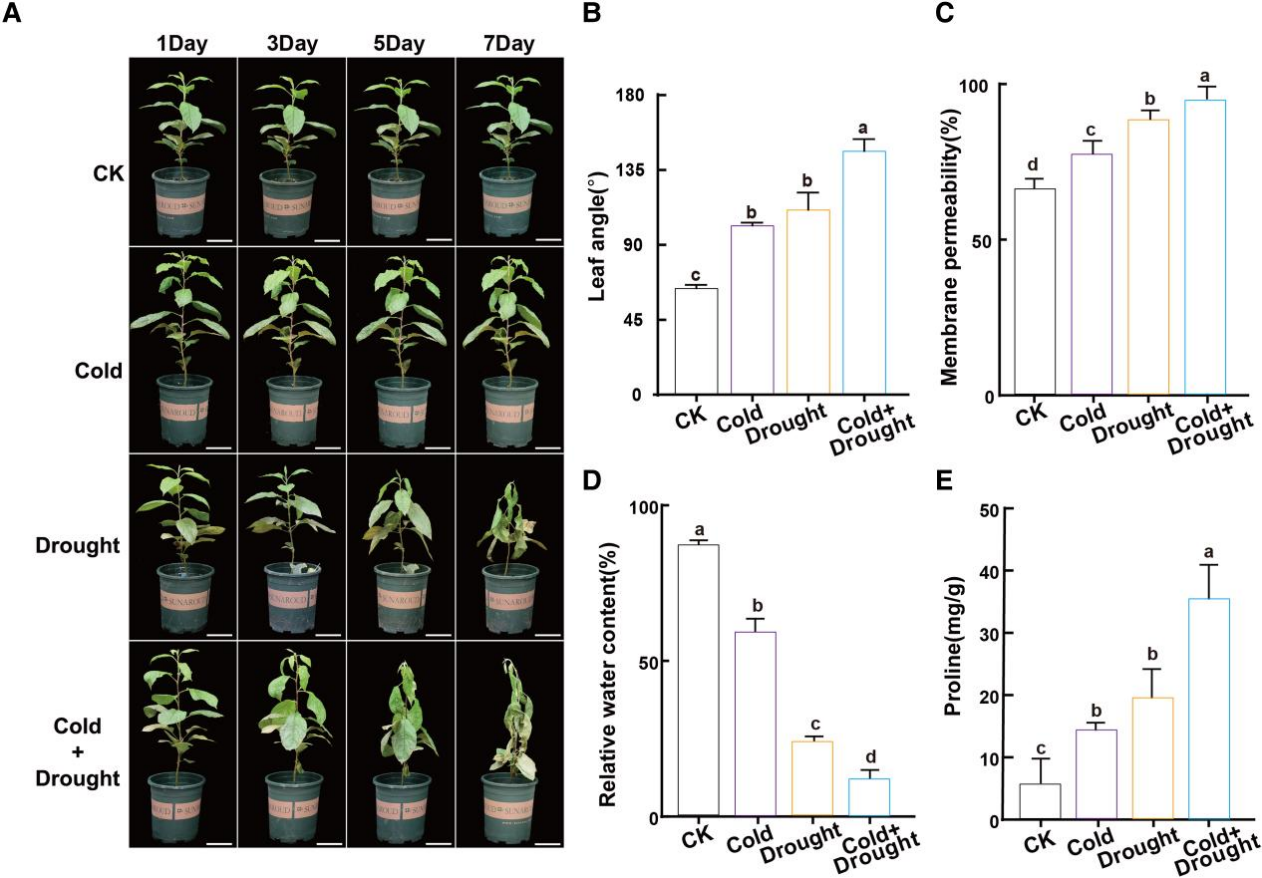
The domesticated Napple rootstock G935 is sensitive to shoot shriveling under drought and cold stress conditions. **A)** Phenotypes under drought, cold, and combined stress treatments. Scale bars, 50 mm. **B** to **E)** Petiole angle **B)**, cell membrane permeability **C)**, relative water content **D)**, and proline content **E)** of apple seedling leaves on the fifth day of stress treatment. CK, non-stressed conditions. Data are means of three replicates ± Sd. Different lowercase letters indicate significant differences according to Duncan’s multiple range test (*P* < 0.05).

To investigate shoot-shriveling damage to apple plants imposed by drought and cold stress, we subjected 3-mo-old plants of the sensitive domesticated apple (*Malus domestica*) rootstock ‘G935' to stress treatments in the laboratory. After 7 d of treatment, apple plants were more stressed under combined (drought + cold) stress than under drought or cold stress alone, as indicated by the degree of leaf wilting and drooping (Fig. 1A). The petiole angle of plants growing under normal, non-stressed conditions (CK) was 60° to 65°, while those of plants under single or combined stress became larger with the extension of stress duration. On the fifth day of treatment, the mean petiole angle of plants under drought stress was about 110°, that of plants under cold stress was approximately 100°, while that of the combined stress plants was over 140° (Fig. 1B). Furthermore, after 5 d of combined stress treatment, the membrane permeability of apple seedling leaves was more than 90%, significantly higher than that of CK or single-stress plants (Fig. 1C). Moreover, the relative water content of apple seedlings under combined stress was only about 13%, which was significantly lower than that in other treatments (Fig. 1D). The proline content of seedling leaves was over 30 mg g^−1^ under combined stress, significantly higher than under drought or cold stress alone, showing a stronger stress response. These results suggest that shoot shriveling caused by both drought and cold stresses involves more serious cell damage, water imbalance, and withering than that caused by single drought or cold stress.

### *MhZAT10* expression responds strongly to combined stress in tolerant apple rootstock

**Figure 2.**
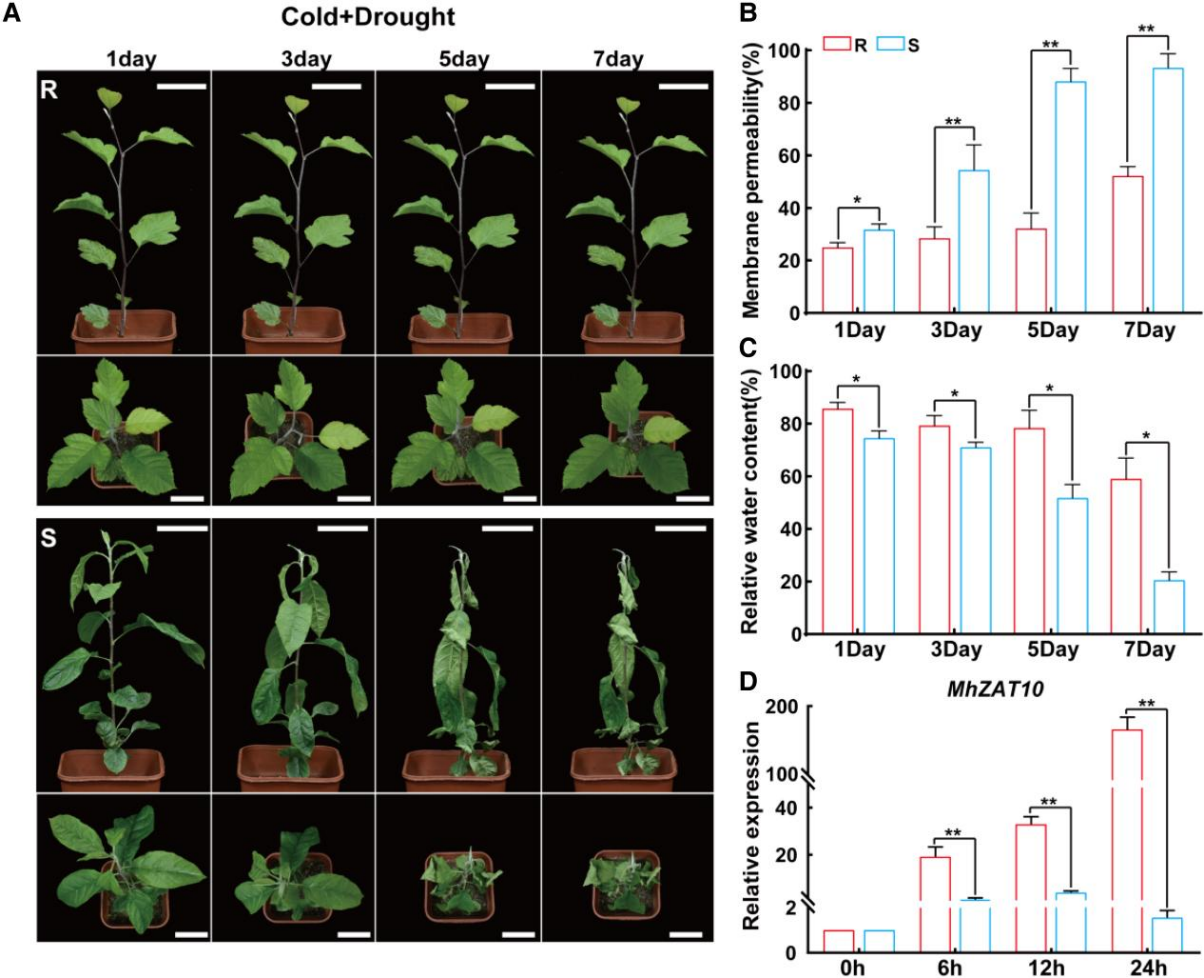
Shoot shriveling in tolerant (SH6) and sensitive (G935) apple rootstocks under drought and cold stress. **A)** Shoot shriveling phenotypes of tolerant rootstock (R) and sensitive rootstock (S). Scale bars, 45 mm. **B** to **D)** Cell membrane permeability **B)**, relative water content **C)**, and transcription levels of *MhZAT10* as detected by RT-qPCR **D)**. Data are means of three replicates ± Sd. Statistical significance was determined using Sidak's multiple test: *P < 0.05; **P < 0.01.

To investigate the mechanism of tolerance to apple shoot shriveling, we compared the tolerant rootstock SH6 (R) with the sensitive rootstock G935 (S). Under drought and cold combined stress, S rootstock showed leaf curling from the third day onward and drooped completely on the seventh day, which was associated with cell membrane permeability increasing to more than 80% and relative water content decreasing to 20% (Fig. 2, A to C). By contrast, R rootstock displayed no major change in phenotypic appearance within 7 d of treatment, cell membrane permeability remained below 50%, and the relative water content was over 60%. We also observed differences in tolerance between R and S rootstocks under single drought or cold stress condition, as indicated by plant growth status and physiological indicators (Supplemental Fig. S1).

We subsequently performed transcriptome deep sequencing (RNA-seq) on R rootstock under drought or cold stress. Drought and cold stress induced 2,475 and 5,252 differentially expressed genes (DEGs), respectively, relative to control R rootstock, of which 1,000 genes responded to both drought and cold stresses (Supplemental Fig. S2). Gene Ontology (GO) term enrichment analysis of these genes revealed 388 DEGs enriched in “membrane components,” “redox process,” “transcriptional regulation,” “metal ion binding,” and other biological processes, including genes encoding 11 types of TFs. Among the TF genes, all six *ZAT* genes actively responded to drought and cold stress, indicating that the *ZAT* gene family might play an important role in enabling crosstalk between drought and cold stress responses. Expression analysis using reverse transcription quantitative PCR (RT-qPCR) showed significantly stronger upregulation of *MhZAT10* (MD08G1086500) in R rootstock under drought, cold, or combined stress than in S rootstock (Fig. 2D, Supplemental Fig. S1, E and H).

### Overexpression of *MhZAT10* enhances drought and cold tolerance in apple rootstock

**Figure 3.**
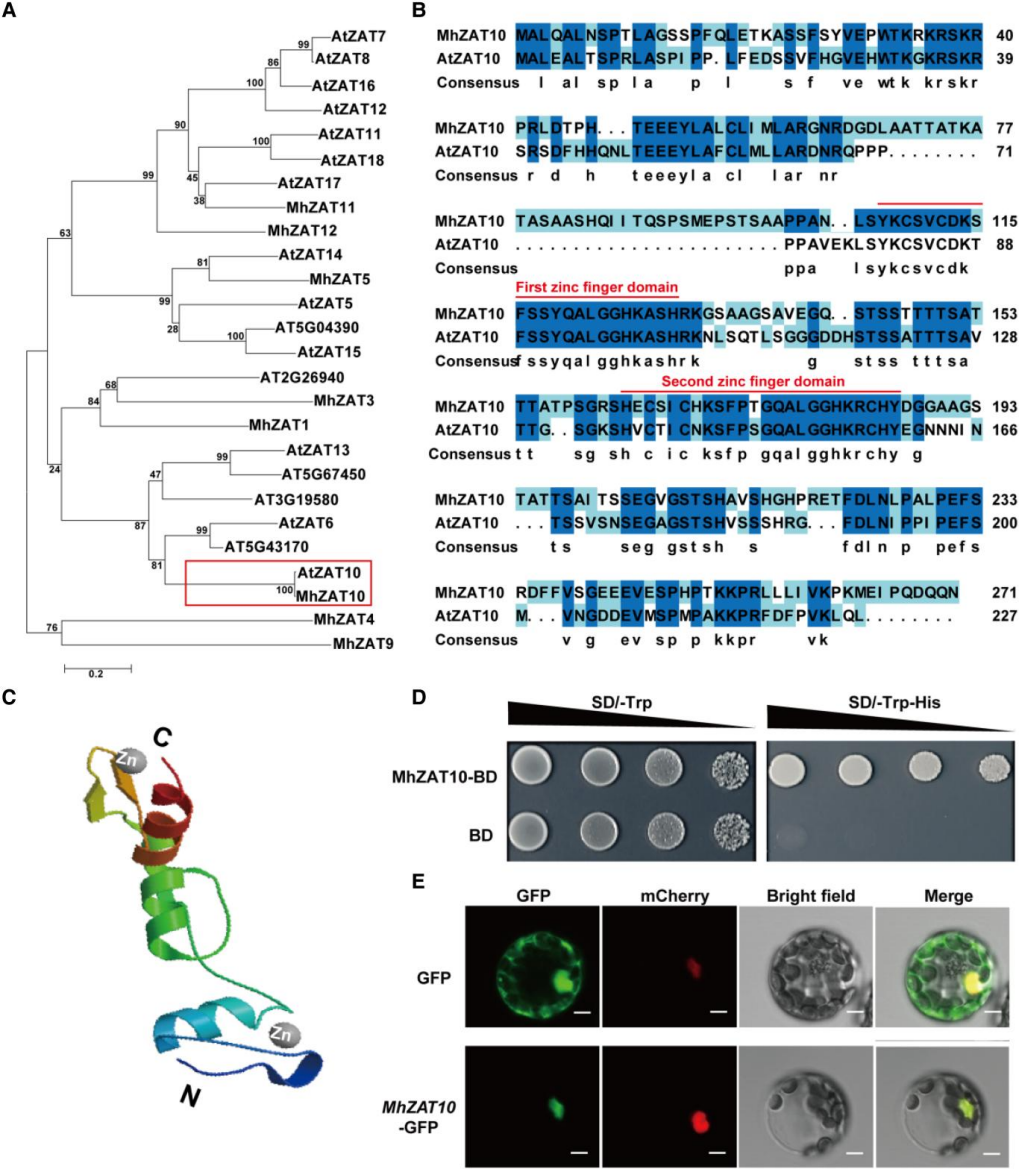
Phylogenetic placement, transcriptional activation, and subcellular localization of MhZAT10. **A)** Phylogenetic analysis of ZAT family protein sequences from apple and Arabidopsis. The numbers on the tree indicate bootstrap supports. The branch lengths were drawn to scale; the bar size indicates the number of amino acid substitutions per site. **B)** Alignment of amino acid sequence between MhZAT10 and AtZAT10. Conserved motifs are marked with straight lines. **C)** Protein structure homology modeling of MhZAT10 predicted using Swiss-Model. The structures in light blue, green, and reddish brown refer to α-helix, yellow–green arrow and yellow arrow refer to β-pleated sheet, and dark blue refers to β-turn. **D)** Transcriptional activation assay of MhZAT10. Each colony was suspended in 10 μL sterile water and then diluted from 10^−1^ to 10^−4^. **E)** Subcellular localization of MhZAT10 in Arabidopsis protoplasts. Scale bars, 10 μm.

Phylogenetic analysis of ZAT family proteins obtained from the Arabidopsis and apple genome databases showed that MhZAT10 and AtZAT10 are closely related (Fig. 3A). We cloned the complete sequence of *MhZAT10* from apple rootstock SH6 and aligned its predicted amino acid sequence to that of AtZAT10 (Fig. 3B). *MhZAT10* encodes a 271-amino acid protein that features two conserved C2H2 zinc finger domains, each containing a conserved QALGGH DNA-binding motif. Protein structure homology modeling predicted that MhZAT10 contains three α-helix regions and can bind two zinc ion ligands (Fig. 3C). To test whether MhZAT10 acts as a transcriptional activator, we cloned the full-length *MhZAT10* sequence into the pGBKT7 vector to create a fusion with the yeast GAL4 DNA-binding domain (GAL4-BD). Growth phenotypes of the resulting transformed yeast colonies indicated that MhZAT10 can activate the *HIS* reporter gene for survival on a synthetic defined (SD) medium lacking Trp and His (SD –TH) (Fig. 3D). To confirm the subcellular localization of MhZAT10, we constructed a plasmid encoding a MhZAT10-green fluorescent protein (GFP) fusion protein and transfected it into Arabidopsis protoplasts. We determined that MhZAT10 localizes to the nucleus, as might be expected given the presumed function of MhZAT10 as a TF (Fig. 3E).

**Figure 4.**
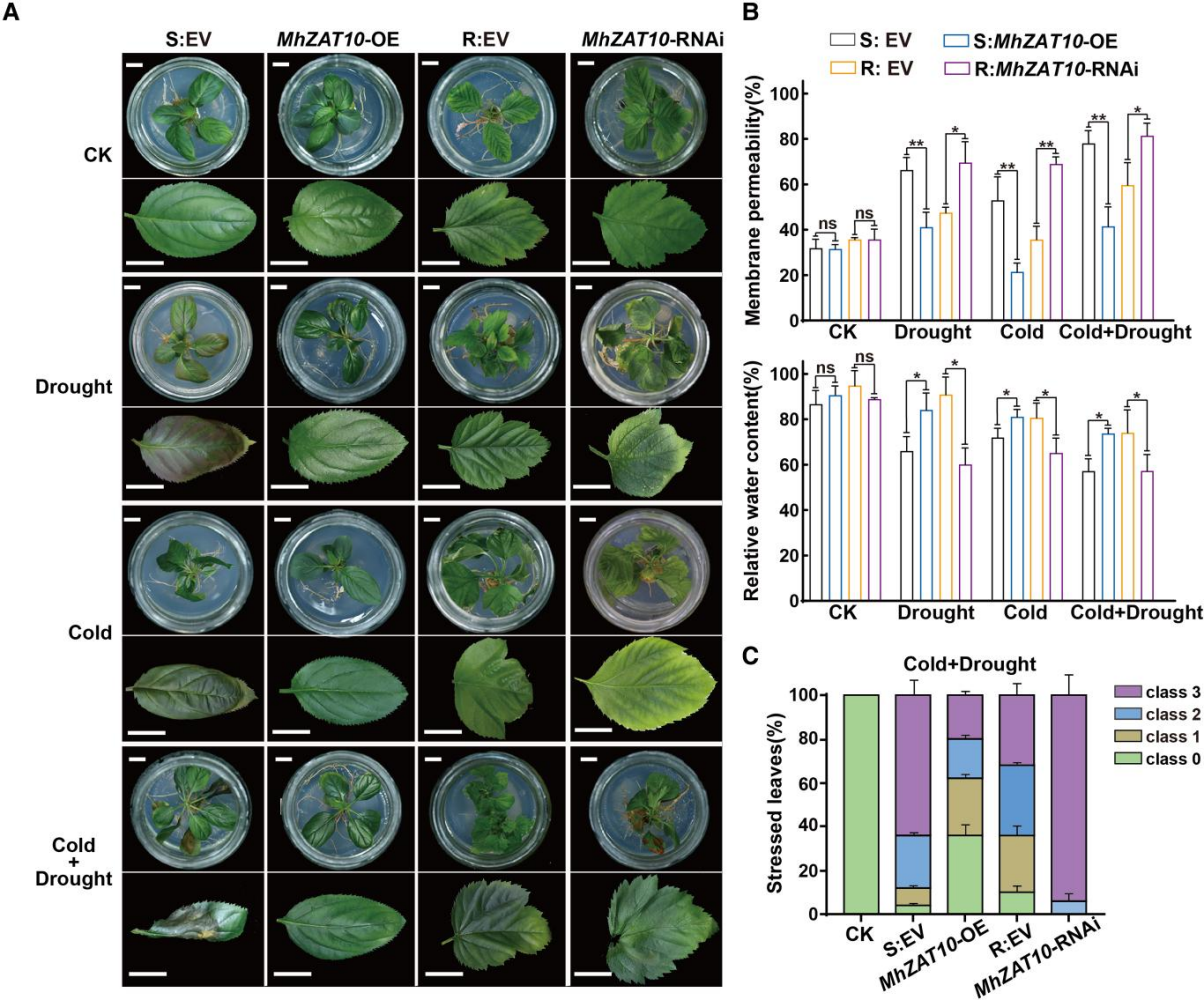
MhZAT10 promotes drought and cold tolerance in apple rootstocks. **A)** Phenotypes of transgenic apple plants heterologously expressing *MhZAT10* in the G935 (S) rootstock (*MhZAT10*-OE), displaying RNA interference (RNAi) in the SH6 (R) rootstock, or harboring empty vector (EV) as controls in the respective rootstocks (S:EV and R:EV) on the seventh day of drought and/or cold conditions. Scale bars, 10 mm. **B)** Cell membrane permeability and relative water content of transgenic plants under stress treatments. **C)** Percentage of stressed leaves showing various degrees of severity of stress injury. Class 0, no symptoms; Class 1, leaf area with injury <20%; Class 2, 20% to 40% leaf area showing injury; Class 3, >40% leaf area showing injury. S, sensitive rootstock; R, tolerant rootstock; CK, non-stressed conditions. All data shown are means of three replicates ± Sd. Statistical significance was determined using Sidak's multiple test: n.s., *P* > 0.05; *P < 0.05; **P < 0.01.

To gain more insight into the function of MhZAT10 in drought and cold tolerance, we used *Agrobacterium rhizogenes* infection and transformation to generate transgenic apple plants heterologously expressing *MhZAT10* in the G935 (S) rootstock (*MhZAT10-*OE), *MhZAT10* RNA interference lines in the SH6 (R) rootstock (*MhZAT10-*RNAi), and empty vector lines as controls in the respective rootstocks (S:EV and R:EV). *MhZAT10* transcript levels were more than six times higher in *MhZAT10-*OE plant roots than in control roots but were nearly undetectable in *MhZAT10-*RNAi plant roots (Supplemental Fig. S3A). None of the transgenic plants showed any abnormality in growth or morphology under normal conditions of in vitro culture (Fig. 4A). However, when plants were grown under drought, cold, or combined stress (drought + cold) for 7 d, S:EV plants displayed obvious curling, wilting, and necrosis in leaves, whereas *MhZAT10-*OE plants exhibited milder symptoms. Furthermore, *MhZAT10-*RNAi plants displayed more severe stress damage than R:EV plants under all three treatments, both in terms of leaf appearance and plant growth. In addition, plants with heterologous expression of *MhZAT10* showed significantly lower cell membrane permeability and elevated relative water content of leaves under each stress treatment compared to those of S:EV plants. In contrast to R:EV plants, *MhZAT10-*RNAi plants suffered more severe damage to cell integrity and greater water loss (Fig. 4B), with a greater percentage of stressed leaves under both combined and single stresses (Fig. 4C, Supplemental Fig. S4A). These results indicate that MhZAT10 positively regulates drought and cold tolerance in apple rootstock.

### The response of MhZAT10 to drought stress depends on the DREB reaction mechanism

To explore the upstream regulatory mechanism of MhZAT10, we cloned the promoter sequence of *MhZAT10* and identified multiple *cis*-elements involved in abiotic stress and phytohormone regulation (Supplemental Fig. S5). Among these regulatory elements, we noticed one copy of DRE, a binding motif for DREB transcription factors involved in drought and cold stress responses. Building on previous results showing a close association between the mRNA abundance of *MhZAT10* and that of *MhDREB2A* (Li et al. 2018), we hypothesized that MhDREB2A might be a potential TF upstream of *MhZAT10*.

**Figure 5.**
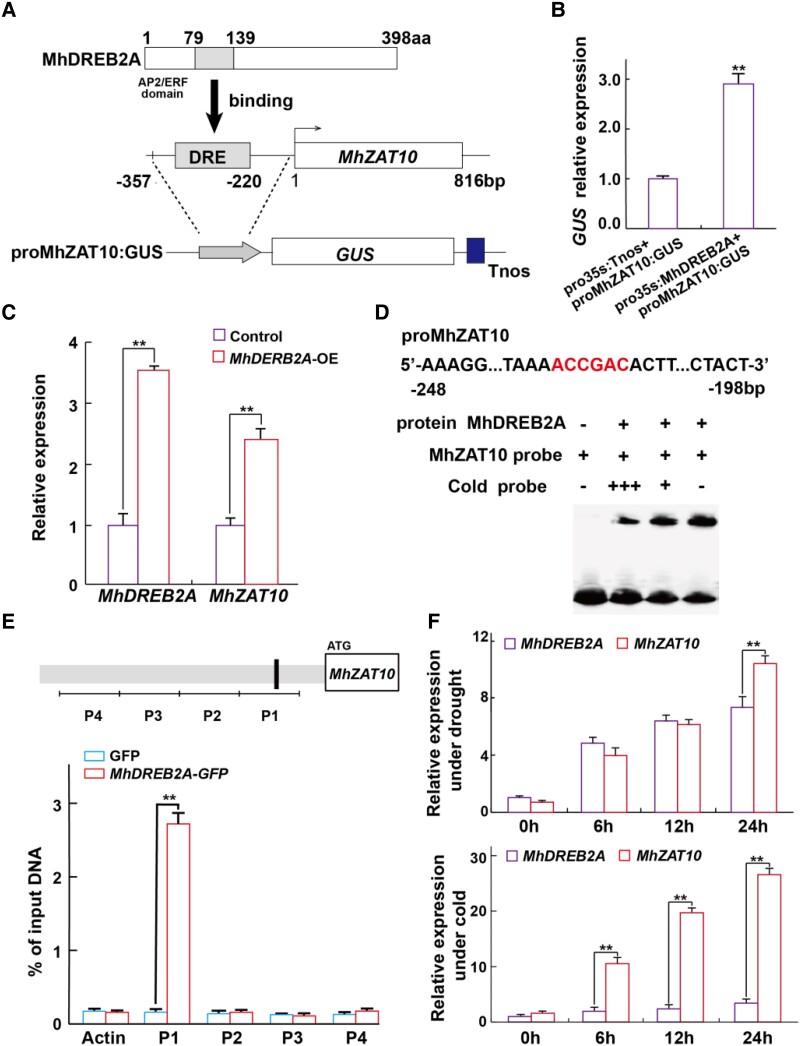
MhDREB2A binds to the *MhZAT10* promoter and activates its expression. **A)** Schematic diagram of MhDREB2A binding to the *MhZAT10* promoter and construction of the *GUS* reporter vector. **B)***GUS* expression in calli from fruit of the apple R rootstock showing that MhDREB2A activates the *MhZAT10* promoter. **C)***MhZAT10* and *MhDREB2A* expression in apple fruit calli (R rootstock) overexpressing *MhDREB2A*. **D)** Electrophoretic mobility shift assay revealing that MhDREB2A binds to the *MhZAT10* promoter. DRE is a 6-bp sequence (5'-ACCGAC-3'). **E)** ChIP-qPCR analysis of MhDREB2A binding to the *MhZAT10* promoter. P1 to P4 indicate the position of qPCR detection for the *MhZAT10* promoter. Actin was used as an internal control. **F)***MhZAT10* and *MhDREB2A* expression in R rootstock leaves under drought stress and cold stress. All date shown are means of three replicates ± Sd. Statistical significance was determined using Sidak's multiple test: **P < 0.01.

We subsequently investigated the transcriptional activation and subcellular localization of MhDREB2A, and results indicated that MhDREB2A has transcriptional activity in yeast and localizes to the nucleus of Arabidopsis protoplasts, as expected function for a TF (Supplemental Fig. S6). The detection of a regulatory relationship between MhDREB2A and *MhZAT10* was carried out using a *β-glucuronidase* (*GUS*) reporter assay in calli from fruit of the R rootstock infected with *Agrobacterium tumefaciens* containing *35S:MhDREB2A* and *proMhZAT10:GUS* constructs (Fig. 5, A and B). When *proMhZAT10:GUS* was cotransformed with *35S:MhDREB2A*, we detected a much higher GUS signal than when *proMhZAT10:GUS* was cotransformed with control vector. In addition, overexpression of *MhDREB2A* in fruit calli also enhanced *MhZAT10* transcript levels, further demonstrating the role of MhDREB2A in promoting *MhZAT10* expression (Fig. 5C). Moreover, we investigated the binding of MhDREB2A to the *MhZAT10* promoter in vitro using an electrophoretic mobility shift assay (EMSA) with fragments of the *MhZAT10* promoter containing the DRE motif as a labeled probe and found that recombinant MhDREB2A protein bound to the *MhZAT10* promoter (Fig. 5D). Chromatin immunoprecipitation with quantitative real-time PCR (ChIP-qPCR) and yeast one-hybrid (Y1H) assays also verified that MhDREB2A can bind to the *MhZAT10* promoter and activates *MhZAT10* expression directly, acting as a TF (Fig. 5E, Supplemental Fig. S7).

Besides, DREB2A and ZAT10 TFs are considered to respond strongly to drought and cold stress, respectively. Our results in leaves of apple R rootstock show that both *MhDREB2A* and *MhZAT10* expression respond to drought stress, whereas *MhZAT10* is cold inducible but *MhDREB2A* is not, indicating that the response of *MhZAT10* to drought stress depends on the MhDREB2A-mediated pathway (Fig. 5F). The investigation of DREB2A expression in leaves of S rootstock show that under drought stress, cold, and combined stress, the expression levels of *MhDREB2A* are all significantly lower than those of R rootstock (Supplemental Fig. S8). In addition, comparative analysis of DREB2A amino acid sequence and *ZAT10* promoter sequence between R and S rootstocks, respectively, shows that both DREB2A amino acid and *ZAT10* promoter sequences share more than 90% similarity (Supplemental Fig. S9). However, a single base difference in *ZAT10* promoter sequence from ACCGAC in R rootstock changes to ACCTAC in S rootstock, which is precisely the DRE motif of R rootstock as indicated in Fig. 5A. According to those results, both DREB2A expression abundance and DRE sequence distinction might cause the difference of *ZAT10* response to drought and cold stresses between R and S rootstocks.

### MhZAT10 is the core TF in drought and cold tolerance

**Figure 6.**
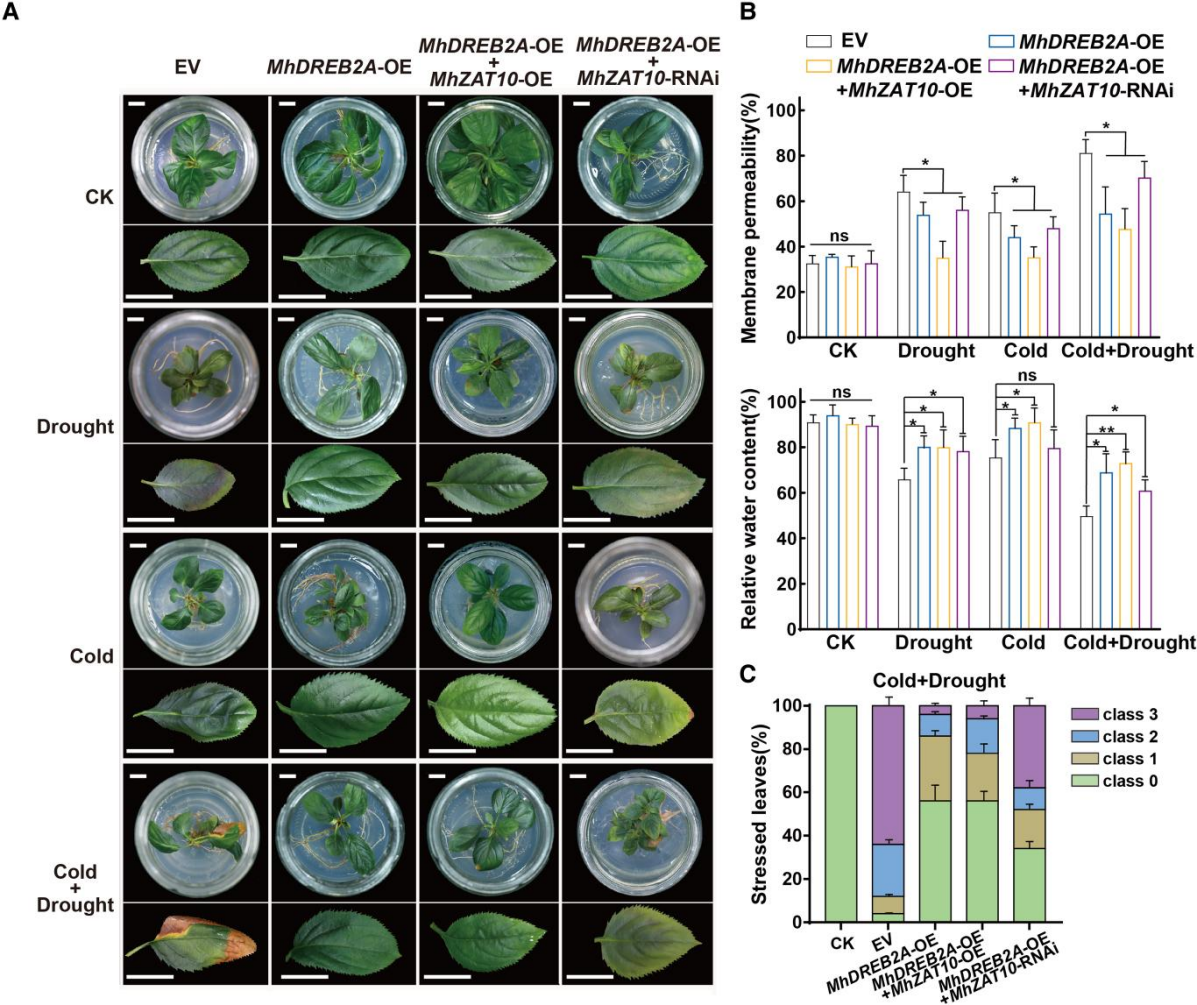
The MhDREB2A*-MhZAT10* module promotes drought and cold tolerance of apple rootstocks. **A)** Phenotypes of transgenic apple plants heterologously expressing *MhDREB2A* and/or *MhZAT10* (*MhDREB2A*-OE, *MhZAT10*-OE), displaying RNA interference (RNAi), or harboring empty vector (EV) as controls in the G935 (S) rootstock on the seventh day of drought and/or cold conditions. Scale bars, 10 mm. **B)** Cell membrane permeability and relative water content of transgenic plants under stress treatment. **C)** Percentage of stressed leaves showing various degrees of severity of stress injury. Class 0, no symptoms; Class 1, leaf area with injury <20%; Class 2, 20% to 40% leaf area showing injury; Class 3, >40% leaf area showing injury. S, sensitive rootstock; R, tolerant rootstock; CK, non-stressed conditions. All data shown are means of three replicates ± Sd. Statistical significance was determined using Sidak's multiple test: n.s., *P* > 0.05; *P < 0.05; **P < 0.01.

To further explore the functions and regulatory relationship between MhDREB2A and MhZAT10 in tolerance to drought and cold stress, we generated four types of transgenic apple plants in the S rootstock with heterologous expression of *MhDREB2A* (*MhDREB2A-*OE), heterologous expression of both *MhDREB2A* and *MhZAT10* (*MhDREB2A-*OE *MhZAT10-*OE), heterologous expression of *MhDREB2A* with RNA interference of *MhZAT10* (*MhDREB2A-*OE *MhZAT10-*RNAi), and empty vector (EV) as control (Fig. 6A). Expression levels of *MhDREB2A* and *MhZAT10* were more than 3-fold different in transgenic OE lines and RNAi lines compared to those in control lines, and we observed promotion of *MhZAT10* expression by MhDREB2A in *MhDREB2A-*OE plants (Supplemental Fig. S3B). We then evaluated the stress tolerance of these transgenic plants to drought, cold, and combined stress. EV plants showed more serious damage under combined stress than under single stress, while phenotypic changes in *MhDREB2A-*OE and *MhDREB2A-*OE *MhZAT10-*OE plants were not obvious. Compared to *MhDREB2A-*OE *MhZAT10-*OE plants, leaves of *MhDREB2A-*OE *MhZAT10-*RNAi plants accumulated red pigments under drought stress, showed curling and yellowing under cold stress, and wilted severely under combined stress, suggesting that functional loss of MhZAT10 weakens plant stress tolerance. Further, physiological measurements revealed that all transgenic plants have significantly lower cell membrane permeability than EV plants, while their relative water content was significantly higher than that of EV plants under each stress treatment. Compared to *MhDREB2A-*OE and *MhDREB2A-*OE *MhZAT10-*OE transgenic plants, however, *MhDREB2A-*OE *MhZAT10-*RNAi transgenic plants displayed higher cell membrane permeability and lower relative water content (Fig. 6B), along with higher severity of stress injury under both combined and single-stress treatments (Fig. 6C, Supplemental Fig. S4B). These results further indicate that although the function of MhDREB2A may not depend solely on the MhZAT10 pathway, the MhDREB2A*-MhZAT10* module plays an important role in improving drought and cold tolerance.

### MhZAT10 induces expression of drought- and cold-tolerant genes

**Figure 7.**
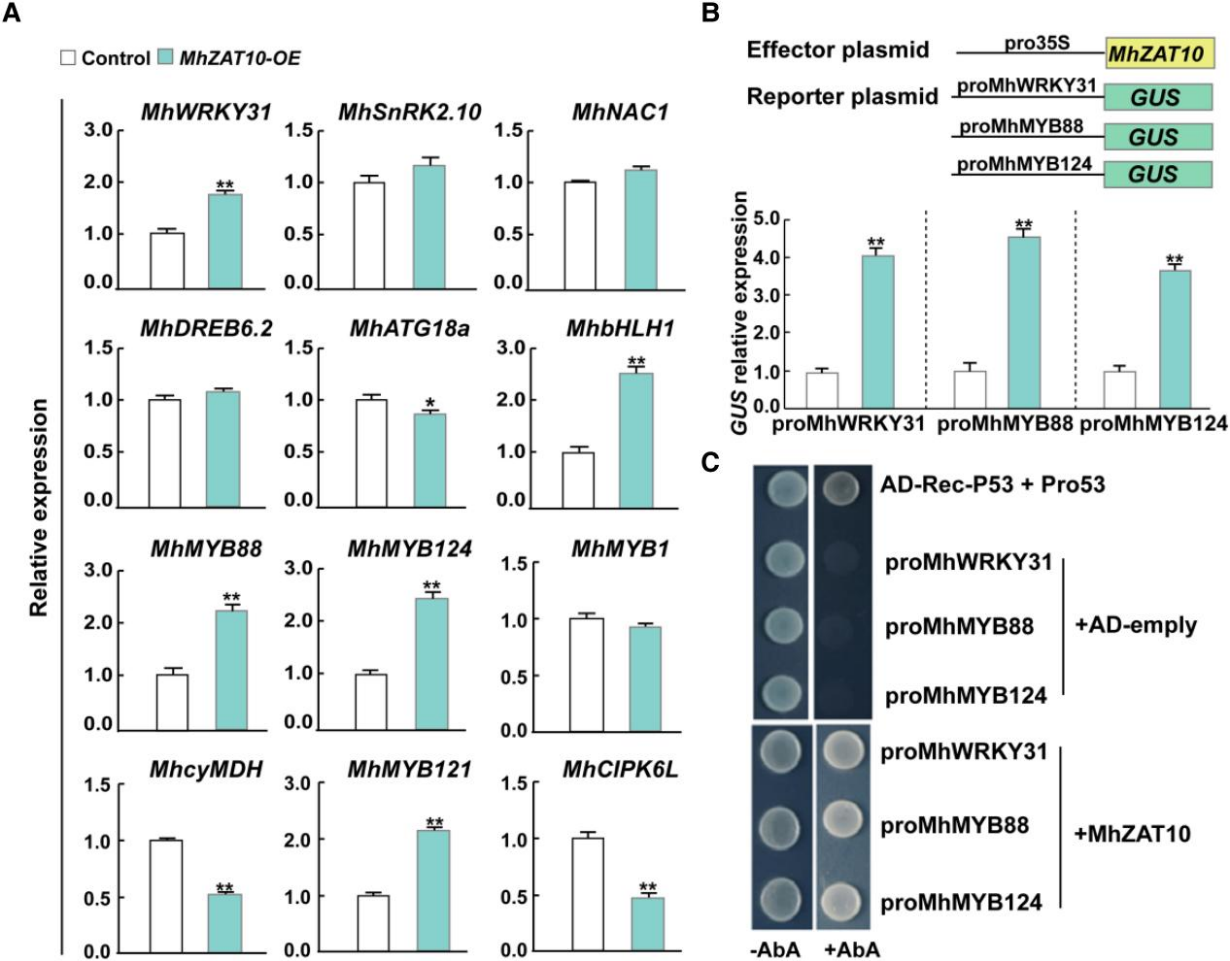
MhZAT10 binds to the *MhWRKY31*, *MhMYB88*, and *MhMYB124* promoters. **A)** Relative expression levels of stress tolerance genes in *MhZAT10-*OE plants. **B)** GUS expression indicating transcriptional activation of the *MhWRKY31*, *MhMYB88*, and *MhMYB124* promoters in apple calli (R rootstock SH6). **C)** Yeast one-hybrid assay showing binding of MhZAT10 to the *MhWRKY31*, *MhMYB88*, and *MhMYB124* promoters. AbA, aureobasidin A. All date shown are means of three replicates ± Sd. Statistical significance was determined using Sidak's multiple test: *P < 0.05; **P < 0.01.

To understand the downstream regulatory mechanism of MhZAT10, we evaluated the expression patterns of genes related to stress tolerance in *MhZAT10-*OE plants by RT-qPCR. We selected five genes associated with drought tolerance [*WRKY31*, *Sucrose non-fermenting-related kinase 2.10* (*SnRK2.10*), *NAC1*, *DREB6.2*, and *AUTOPHAGY-RELATED 18a* (*ATG18a*)], three genes associated with cold tolerance [*basic helix-loop-helix 1* (*bHLH1*), *MYB88*, and *MYB124*], and four genes associated with both cold and drought double tolerance [*cytosolic NAD-dependent malate dehydrogenase* (*cyMDH*), *MYB1*, *MYB121*, and *CBL-interacting protein kinase 6* (*CIPK6L*)] that had been identified previously and validated in transgenic *Malus* lines. Of these 12 genes, five (*MhWRKY31*, *MhbHLH1*, *MhMYB88*, *MhMYB121*, and *MhMYB124*) were upregulated in *MhZAT10-*OE plants (Fig. 7A). To identify potential regulatory targets of MhZAT10, we performed a *GUS* reporter assay in apple calli (R rootstock SH6). MhZAT10 activated transcription from *MhWRKY31*, *MhMYB88*, and *MhMYB124* promoters (Fig. 7B). Furthermore, Y1H assays also supported MhZAT10 binding to these promoters (Fig. 7C). Our results demonstrate that drought-tolerant *MhWRKY31* and cold-tolerant *MhMYB88* and *MhMYB124* are target genes of the TF MhZAT10.

## Discussion

The growth, development, and yield of plants are often limited by adverse natural environments. In the production of woody fruit trees, especially, the combined stress of drought and cold is the main cause of shoot shriveling, resulting in a large reduction in fruit yield. Many reports have described the effects of individual drought or cold stress on plant growth and development, such as changes in the cytoskeleton, phytohormone levels, and metabolism (Sangwan et al. 2001; Achard et al. 2008; Geng et al. 2020). Distinct from previous studies, we focused on the combination of drought and cold stresses on plant growth, which we demonstrated were more harmful to apple plants than single stress. Loss of leaf relative water content was more severe and more often lethal to plants subjected to combined stress and caused shriveling (Fig. 1). In addition, we also detected twice as many cold-induced DEGs than drought-induced DEGs, with cold stress inducing the expression of approximately 50% of all drought response genes as shown in the present study (Supplemental Fig. S2) and our previous study (Li et al. 2019b). Therefore, apple plants appear to reprogram their transcriptome when exposed to cold stress to a greater extent than in response to drought stress.

The functions of C_2_H_2_ transcription factors have been well characterized in Arabidopsis, and most members of the C_2_H_2_ zinc finger protein family are involved in abiotic stress responses, such as ZAT7, ZAT10, and ZAT12 (Kie Bowicz-Matuk 2012; Nguyen et al. 2012; Liu et al. 2017). ZAT10 and ZAT12 act independently of the ICE-CBF-COR pathway; ZAT12 inhibits the expression of *CBF1–3*, appears to be involved in negative regulation of the CBF cold response pathway, and plays a role upstream of ZAT10 through the MAPK signal transduction pathway (Heidarvand and Amiri 2010; Park et al. 2015; Nguyen et al. 2016). ZAT10 was initially identified as a salt- and cold-reactive protein, responding to cold stress and inducing expression of *COR* genes under cold stress (Ding et al. 2019; Liu et al. 2022). However, ZAT10 also inhibits the expression of the abiotic stress defense gene *RESPONSIVE TO DESICCATION 29A* (*RD29A*), which acts downstream of CBF3 (Sakamoto et al. 2000; Sakamoto et al. 2004). These results suggest that C_2_H_2_ zinc finger proteins are required for stress tolerance and possibly play a dual role as both activators and repressors of stress response genes. Interestingly, transgenic plants with constitutive expression or even knockout of *ZAT10* are more tolerant to drought stress, osmotic stress, and salt and heat stresses than wild-type plants (Mittler et al. 2006; Xiao et al. 2009; He et al. 2019), while others display reduced tolerance to drought stress (Sakamoto et al. 2004; Yang et al. 2021), suggesting that positive or negative effects on stress tolerance are associated with ZAT10 species or sequence specificity.

We characterized *MhZAT10* from drought- and cold-tolerant apple rootstock using RNA-seq and observed significantly stronger upregulation of *MhZAT10* in tolerant rootstock than in sensitive rootstock under drought, cold, or combined stress (Fig. 2, Supplemental Fig. S2). This result indicates that MhZAT10 participates in the drought and cold stress responses simultaneously, with a degree of crosstalk in the complex underlying regulatory network. To clarify the positive regulatory function of MhZAT10 in tolerant apple rootstock, we designed a complementary functional verification test, in which *MhZAT10* expression was disrupted in tolerant rootstock through heterologous RNAi-mediated silencing and enhanced through overexpression in sensitive rootstock. Ectopic expression of *MhZAT10* enhanced the tolerance of sensitive apple rootstock to drought and cold stress, whereas knockdown of *MhZAT10* reduced the stress tolerance of tolerant apple rootstock (Fig. 4). Thus, we confirmed the positive functions of MhZAT10 in response to drought and cold stress in apple rootstock.

To achieve functional verification of *MhZAT10* in apple rootstock, we established a genetic transformation method by the application of *A. rhizogenes* strain MSU440 on the rootstock SH6 and G935. As reported previously, *A. rhizogenes* can be used to induce adventitious roots named “hairy roots’ upon wounding and infection of plant stems, and a target gene can be transferred and incorporated into the genome of the host plant, resulting in transgenic hairy roots (Meng et al. 2019; Cao et al. 2023). One of the benefits of “composite” plants consisting of wild-type shoots and transgenic roots is that the effects of transgenic roots on the up-ground wild-type leaves, flowers, stem, or fruits can be observed in gene function studies, such as high-salt tolerance gene *TaNHX2* in soybean (*Glycine max*) (Cao et al. 2011) and drought tolerance gene *CcCIPK14* in pigeon pea (*Cajanus cajan*) (Meng et al. 2021). As an organic whole, root activity and characteristics have a direct impact on plant growth and development, including tree vigor, fruit yield and quality, and stress resistance, especially for fruit tree rootstocks when the same variety is grafted on different rootstocks (Noda et al. 2000; Obermiller and McArtney 2011; Li et al. 2017a). For investigating the drought and cold tolerance of *MhZAT10* in apple rootstock, we mainly observed the wilting degree of leaves, namely the water supply ability of transgenic roots under drought and cold stresses, which is exactly the key reason of shoot shriveling. In view of those results of leaf phenotypes for *MhZAT10* overexpression or knockdown “composite” plants, we defined the tolerance function of *MhZAT10* and also revealed the applicability of *A. rhizogenes* to a certain extent for gene function research in apple rootstock.

How does MhZAT10 regulate the stress response of plants to water shortage? Further exploration of *cis*-elements in the *MhZAT10* promoter region identified a DRE element. ABA-dependent and ABA-independent stress response genes can be activated by drought stress. Gene expression in the ABA-dependent pathway is activated through the ABA responsive element (ABRE), while gene expression in the ABA-independent stress response is regulated through the DRE element (Shinozaki and Yamaguchi-Shinozaki 2007; Yoshida et al. 2014). During cold signal transduction, the ABA-independent TF DREB1 recognizes DRE/CRT elements, and transgenic plants overexpressing *DREB1* show enhanced tolerance to cold stress (Yang et al. 2011; Fernando 2018; Moon et al. 2019). DREB2A and DREB2B are highly induced under osmotic stress and transactivate the DRE *cis*-element of stress response genes, mainly under drought or salt stress (Nakashima et al. 2009; Dhriti and Ashverya 2015; Meena et al. 2022). In *Malus*, a total of 68 putative DREBs were found, from which 16 members are DREB2s (Zhao et al. 2012). *DREB2A* is expressed mainly in apple roots and leaves and dramatically induced by drought, high salt, and ABA stresses in *Malus domestica* (Li et al. 2017b), *Malus zumi* (Cao et al. 2019), *Malus prunifolia* (Li et al. 2019a), and *Malus hupehensis* (Dang et al. 2022). *DREB2A* transgenic apple calli, Arabidopsis, and *Nicotiana benthamiana* initiate the transcriptional regulation of genes associated with plant growth and cell cycle, cause the synthesis of osmotic regulation substances such as proline and sucrose, and enhance the resistance of plants to drought, high salt, and other environmental stresses, comprehensively improving plant agronomic traits (Li et al. 2019b; Dang et al. 2022). However, the exploration of *DREB2A* involving in cold signal transduction in apple and evaluation of transgenic apple plants on cold tolerance were rarely carried out.

In this study, we showed that *MhZAT10* responded to both drought and cold stresses, with mRNA levels exhibiting a close association with *MhDREB2A*. Cotransformation of *proMhZAT10:GUS* and *35S:MhDREB2A* into apple calli resulted in higher *GUS* expression than cotransformation of *proMhZAT10:GUS* with a vector control; heterologous expression of *MhDREB2A* in apple calli also enhanced the expression of endogenous *MhZAT10*. EMSA, ChIP-qPCR, and Y1H assays demonstrated that MhDREB2A associates with the *MhZAT10* promoter (Fig. 5, Supplemental Fig. S7). These results show that *MhZAT10* is regulated by MhDREB2A. We also characterized the function of the MhDREB2A*-MhZAT10* module in stress signaling in vivo. Heterologous expression of both *MhDREB2A* and *MhZAT10* enhanced the tolerance of apple rootstocks to combined stress compared to that of control plants, while heterologous expression of *MhDREB2A* and inhibition of *MhZAT10* provided less tolerance (Fig. 6). Therefore, we revealed that MhZAT10 plays a key role in dual stress signal transduction and is a core integrator of responses to drought and cold stresses.

We identified 12 downstream target genes of MhZAT10 involved in drought and cold tolerance whose involvement in drought and/or cold tolerance has previously been verified by transgene studies in apples. Five of these 12 genes were upregulated, and three genes were downregulated in *MhZAT10-*OE plants, revealing the dual role of MhZAT10 as both an activator and a repressor of stress response genes (Fig. 7). Furthermore, *GUS* reporter and Y1H assays showed that MhZAT10 bound to the promoters of *MhWRKY31*, *MhMYB88*, and *MhMYB124* to activate their transcription, confirming that drought-tolerant *MhWRKY31* and cold-tolerant *MhMYB88* and *MhMYB124* were target genes of the TF MhZAT10. *MdMYB88* and *MdMYB124* are cold stress–induced genes in apples, and transgenic apples overexpressing *MdMYB88* and *MdMYB124* show enhanced tolerance to cold stress (Xie et al. 2018). Under cold stress induction, MdMYB88 and MdMYB124 activate *CIRCADIAN-CLOCK ASSOCIATED 1* (*MdCCA1*) and regulate the response of *MdCBF* genes, inducing *COR* gene expression. In addition, MdWRKY31 is a key factor of ABA-dependent drought tolerance and further activates downstream drought resistance related (DRR) genes (Zhao et al. 2019). Therefore, our results verified the involvement of the MhDREB2A-*MhZAT10* module in tolerance to both drought and cold stresses by regulating the expression of *MhWRKY31*, *MhMYB88*, and *MhMYB124*.

**Figure 8.**
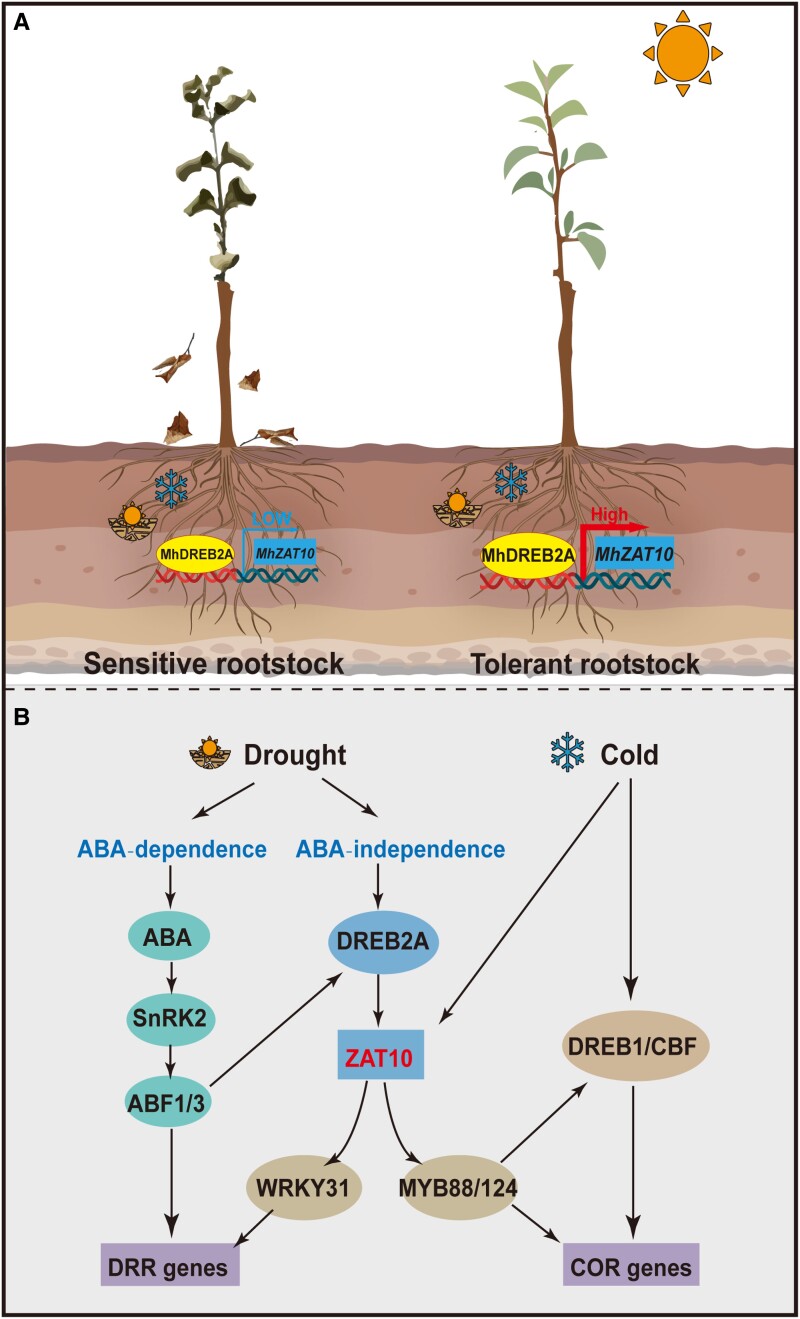
Proposed working model for the MhDREB2A-*MhZAT10* module in drought and cold stress responses in apple. **A)** Shoot shriveling–sensitive and shoot shriveling–tolerant apple rootstocks under drought and cold stress. **B)** Mechanism of MhDREB2A-*MhZAT10* response to drought and cold stress. MhZAT10 responds to drought stress independently of the ABA signaling pathway, being directly regulated by MhDREB2A, and responds to cold stress independently of the CBF pathway. MhZAT10 also directly positively regulates expression of *MhWRKY31* and *MhMYB88*/*124* genes, which promote drought and cold tolerance, respectively, via ABA-independent drought signaling and the CBF-dependent and CBF-independent cold signaling pathway.

Taking our results together with those of previous studies, we propose a working model for the function of MhDREB2A-*MhZAT10* in response to drought and cold stress in apple (Fig. 8). Under drought and cold combined stress, MhZAT10 of rootstock showing tolerance to shoot shriveling responded more strongly than that of the sensitive rootstock, suggesting that ZAT10 might be a key regulator of stress tolerance in apple. Specifically, a drought signal leads to activation of ABA-independent pathways, in which *MhDREB2A* is induced upstream of *MhZAT10* and its encoding protein directly regulates *MhZAT10* expression by binding to the DRE motif of its promoter. At the same time, *MhZAT10* expression is also positively regulated by cold stress. MhZAT10 then modulates the drought response gene *MhWRKY31* and the cold response genes *MhMYB88* and *MhMYB124* to regulate plant tolerance, further inducing *DRR* and *COR* gene expression. Thus, MhDREB2A-*MhZAT10* senses drought and cold stress as a whole and plays a critical role in crosstalk between drought and cold stress responses to improve shoot shriveling tolerance in apple.

## Materials and methods

### Plant material and stress treatment

Apple rootstock ‘SH6' (*Malus honanensis*) and ‘G935' (*Malus domestica*) cuttings of about 3 mo of age and showing uniform development were collected and cultured in a growth chamber (25 °C, 150 μmol photons m^−2^ s^−1^, relative humidity 60% to 65%, and 16-h light photoperiods). Before stress treatments, potted cutting plants were irrigated every 3 d, and after the plants were stable for a week in the growth chamber, drought stress was induced by withholding water and cold stress and combined stress (drought + cold) were carried out by adjusting the chamber to 4 °C. The plants used for drought and combined stress treatment were stopped being watered, while the plants under cold stress were continued to be watered. In vitro plantlets of the apple rootstocks were cultured on Murashige and Skoog (MS) medium supplemented with 0.6 mg L^−1^ 6-benzylaminopurine (6-BA) and 0.4 mg L^−1^ 3-indolebutyric acid (IBA) at 25 °C under 16-h light photoperiods. Healthy and uniform in vitro plantlets of the apple rootstocks were used for stress treatments and were grown in medium supplemented with 1 M mannitol for simulated drought stress at 25 °C and cold and combined stress treatments at 4 °C. All treatments lasted for 1 wk and were performed in triplicate using at least six plants for each replicate. SH6 fruit calli were induced and cultured on MS medium supplemented with 1.0 mg L^−1^ 2,4-dichlorophenoxyacetic acid (2,4-D) and 0.1 mg L^−1^ 6-BA at 25 °C in the dark and subcultured every 4 wk.

### RNA-seq assays

Leaves of SH6 plants under drought and cold stress were collected separately on the fifth day in liquid nitrogen. Biomarker Technologies Corporation (Beijing, China) extracted total RNA and performed RNA-seq with three biological repeats. The percentage of *Q*_30_ in each sample of clean data was >93%. The apple genome (https://iris.angers.inra.fr/gddh13/) was used as reference, and DEGs (false discovery rate [FDR] < 0.01, fold change ≥ 2.0) and GO enrichment pathway were performed in BMKcloud (https://international.biocloud.net/). The RNA-seq data were submitted to National Genomics Data Center (https://ngdc.cncb.ac.cn/bioproject/, China) with BioProject number PRJCA013120.

### RNA extraction and RT-qPCR analysis

Total RNA was extracted using an EASYspin plant RNA extraction kit (Biomed, China), and first-strand cDNAs were synthesized using Moloney murine leukemia virus (M-MLV) reverse transcriptase (Promega, USA) according to the manufacturer's instructions. RT-qPCR was performed on a Bio-Rad C1000 Thermal Cycler with a CFX384 Real Time System (Bio-Rad, USA), using 2× SYBR-Green I RT-qPCR Master Mix (Takara, Japan) as the labeling agent. Three biological replicates and three technical repeats were performed, and relative expression levels in three biological replicates were calculated using the 2^−△△Ct^ method. *β-Actin* (XM008356922) was amplified along with the target genes as an internal control. Sequences of all primers used for RT-qPCR assays are listed in Supplemental Table S1.

### Vector construction and plant transformation

To construct plasmids *MhZAT10*-OE and *MhZAT10*-RNAi, the full-length coding sequence and a specific 480-bp sequence (for antisense) of *MhZAT10* were cloned into the pCAMBIA1304 vector. The full-length sequence of *MhDREB2A* was inserted into the pCAMBIA1304 vector to construct the *MhDREB2A*-OE vector. Promoters of *MhZAT10*, *MhWRKY31*, *MhMYB88*, and *MhMYB124* were inserted into the pCAMBIA1304 vector to construct *proMhZAT10:GUS*, *proMhWRKY31:GUS*, *proMhMYB88:GUS*, and *proMhMYB124:GUS* reporter vectors, respectively. Sequences and restriction sites of all primers used for vector construction are listed in Supplemental Table S2. Agrobacterium (*A. rhizogenes*) strain MSU440 was used for transformation of apple plantlets as previously reported (Meng et al. 2019). Healthy and uniform in vitro plantlets of the apple rootstocks about 2 to 3 cm high were cut and infected by 1 mL sterile syringe containing *Agrobacterium* suspension on the shear surface and sides near the bottom and then transferred to coculture medium (1/2 MS supplemented with 0.5 mg L^−1^ 6-BA) at 22 °C in the dark for 3 d. After washing with sterile water, plantlets were transferred into rooting medium (1/2 MS supplemented with 250 mg L^−1^ cefotaxime and 250 mg L^−1^ Timentin) and cultured at 25 °C under 16-h photoperiods. For SH6 fruit calli transformation, *A. tumefaciens* strain EHA105 was used and processed as described by Li et al. (2019a). Seven-d-old calli was immersed into *Agrobacterium* suspension for 12 min and then was cocultured on MS medium supplemented with 1.0 mg L^−1^ 2,4-D and 0.1 mg L^−1^ 6-BA at 25 °C in the dark for 2 d. Subsequently, the calli was washed three times with sterile water and then stored.

### Subcellular localization assays

The coding sequences of *MhZAT10* and *MhDREB2A* were amplified and subcloned into the pEZS-NL-enhanced green fluorescent protein (EGFP) vector, respectively, resulting in a construct encoding an N-terminal fusion with EGFP. The *MhZAT10-EGFP* and *MhDREB2A-EGFP* constructs were transiently transfected in Arabidopsis protoplasts, respectively (Cao et al. 2016). Nucleus-localized SV40-mCherry was used to identify the nucleus. After a 12-h incubation in W1 solution (0.5 M mannitol, 4 mM MES-KOH, 20 mM KCl, pH 5.7), the protoplasts were imaged using a confocal laser scanning microscope (Olympus FV1000 view, Japan) equipped with an argon ion laser. EGFP fluorescence was visualized by scanning with a 488-nm laser at minimal power level and 505- to 530-nm spectral detection. And excitation wavelength 561 nm and detection wavelength 590 to 650 nm were used for mCherry fluorescence detection. Primers used for vector construction are shown in Supplemental Table S2.

### Transcriptional activation and Y1H assays

For transcriptional activation assays, the coding sequences of *MhZAT10* and *MhDREB2A* were cloned into the pGBKT7 vector, respectively. Empty pGBKT7 was used as a negative control. These constructs were introduced into yeast Y1HGold cells (Clontech, USA). Growth performance of yeast colonies was analyzed on synthetic defined (SD) medium lacking Trp (SD –Trp) or Trp and His (SD –Trp–His). For Y1H assay, the coding sequences of *MhDREB2A* and *MhZAT10* were inserted into the pGADT7 vector, respectively. Sequences of the *MhZAT10*, *MhWRKY31*, *MhMYB88*, and *MhMYB124* promoters were individually inserted into the pAbAi vector. These constructs were cotransformed into yeast cells, and growth performance of yeast was analyzed on SD –Ura, SD –Leu, or SD –Leu + AbA, respectively. Primers used for vector construction are listed in Supplemental Table S2.

### EMSA

The coding sequence of *MhDREB2A* was amplified by PCR and cloned into the pEASY-Blunt E2 vector. The resulting construct (pEASY-Blunt E2-MhDREB2A-His) was transformed into *Escherichia coli* strain Transetta DE3 pLysS. Production and purification of recombinant MhDREB2A-His fusion protein were performed using Ni-NTA agarose (Millipore, USA). Electrophoretic mobility shift assay was performed using a LightShift Chemiluminescent EMSA Kit (Thermo Scientific, USA) according to the manufacturer's instructions. Oligonucleotide probes of *MhZAT10* were synthesized and labeled with 5′-biotin (Sangon Biotech, China). Binding specificity was determined by testing competition with excess unlabeled oligonucleotides. Primers and probes used are listed in Supplemental Table S2.

### ChIP-qPCR analysis

The full-length sequence of *MhDREB2A* was inserted into the pRI101 vector to construct the recombinant vector *35S:MhDREB2A-GFP* and then was transformed into SH6 fruit calli as described above. ChIP analysis was performed using the EpiQuik Plant ChIP Kit (EpiQuik, USA) as the manufacturer's instructions. Calli tissue was treated with formic acid under vacuum for 10 min to cross link DNA and protein, and extracts were incubated with GFP antibody (Transgen Biotech, China) for 2 h at room temperature. DNA released from the antibody-captured protein–DNA complex was retrieved and purified through fast-spin column. Purified immunoprecipitated DNA was used as the template for the qPCR analysis. Each ChIP assay was repeated three times and the enriched DNA fragments in each ChIP sample were used as one biological replicate for qPCR. *β-Actin* was used as an internal control and four regions P1 to P4 of the *MhZAT10* promoter were analyzed to assess their enrichment. Primers used are listed in Supplemental Table S2.

### Determination of cell membrane permeability, relative water content, and proline content

For tolerance assays, the fifth to sixth mature leaves from the top of apple rootstock plants were used for determination of physiological indexes. Determination of relative water content (RWC) was based on the method described by Zhao et al. (2019) as follows: RWC (%) = (fresh weight − dry weight)/(rehydrated weight − dry weight) * 100. Cell membrane permeability was measured using a conductivity meter (Leci, DDS-307), and proline content was determined using the acid ninhydrin procedure as described previously (Wang et al. 2017a).

### Phylogenetic analysis of ZAT10 proteins

Sequences of 26 ZAT10 proteins were obtained from *Malus domestica* (https://iris.angers.inra.fr/gddh13/) and Arabidopsis (*Arabidopsis thaliana*) (https://www.arabidopsis.org/index.jsp) databases. The amino acid sequences of all ZAT10 proteins were analyzed using ClustalW (http://www.clustal.org/clustal2/), and a phylogenetic tree was constructed using the neighbor-joining method in MEGA7.0 software (https://www.megasoftware.net/). Accession numbers and names of ZAT10 proteins are shown in Supplemental Table S3.

### Statistical analyses

All statistical analyses were performed using GraphPad Prism version 8.0 software, and statistically significant differences are denoted with different lowercase letters or with asterisks. Details of the analysis are given in the respective figure legends.

#### Accession numbers

Sequence data from this article can be found in The Apple Genome (https://iris.angers.inra.fr/gddh13/) data libraries or GenBank (NCBI, https://www.ncbi.nlm.nih.gov/) under accession numbers *MhZAT10* (MD08G1086500/OP562895), *MhDREB2A* (XM008355947), *MhWRKY31* (XM008365867.3), *MhSnRK2.10* (KJ868181.1), *MhNAC1* (MF401514.1), *MhDREB6.2* (KX098453.1), *MhATG18a* (KC800804), *MhbHLH1* (NM001294038.1), *MhMYB88* (KY569647.1), *MhMYB124* (KY569648.1), *MhcyMDH* (DQ221207.1), *MhMYB1* (KC691248), *MhMYB121* (KC834015.1), and *MhCIPK6L* (NM001328820.1).

## Supplementary Material

kiad147_Supplementary_Data

## Data Availability

The authors confirm that all experimental data are available and accessible via the main text and/or the supplemental data.

## Supplemental data

The following materials are available in the online version of this article.

**Supplemental Figure S1**. Analysis of shoot shriveling in tolerant (SH6) and sensitive (G935) apple rootstocks under drought and cold stress.

**Supplemental Figure S2**. RNA-seq analysis of shoot-shriveling tolerant rootstock SH6 under drought and cold stress.

**Supplemental Figure S3**. Detection of *MhZAT10* and *MhDREB2A* expression levels in transgenic plants.

**Supplemental Figure S4**. Percentage of stressed leaves showing various degrees of severity of stress injury.

**Supplemental Figure S5**. *Cis*-elements and putative regulating factors in the *MhZAT10* promoter.

**Supplemental Figure S6**. Transcriptional activation assay and subcellular localization of MhDREB2A.

**Supplemental Figure S7**. Yeast one-hybrid assay showing binding of MhDREB2A to the *MhZAT10* promoter.

**Supplemental Figure S8**. Detection of *MdDREB2A* expression levels in shoot-shriveling sensitive rootstock G935 under drought and cold stress.

**Supplemental Figure S9**. Alignment of DREB2A amino acid sequence and *ZAT10* promoter sequence between tolerant (SH6) and sensitive (G935) apple rootstocks.

**Supplemental Table S1**. Primer sequences used for RT-qPCR detection.

**Supplemental Table S2**. Primer sequences used for vector constructions.

**Supplemental Table S3**. ZAT family protein list.
