# Supplementary material for: Transcription factors MhDREB2A/MhZAT10 play a role in drought and cold stress response crosstalk in apple
Source: Plant Physiol. 2023 Mar 6;192(3):2203–20. doi: 10.1093/plphys/kiad147 (PMC10315272; doi:10.1093/plphys/kiad147)
Supplement: kiad147_Supplementary_Data [file kiad147_supplementary_data.pdf]

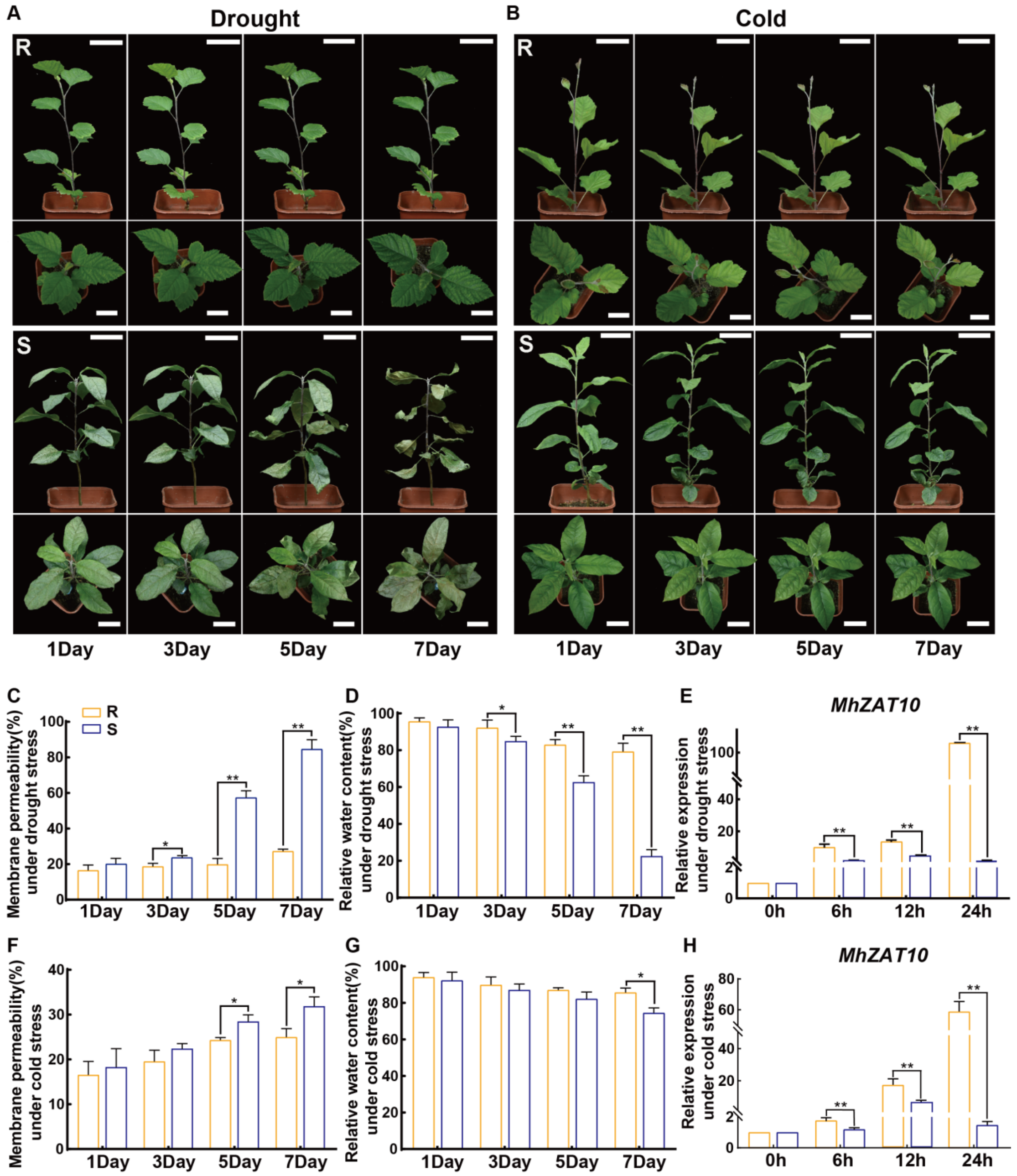

Supplemental Figure S1 Analysis of shoot shriveling in tolerant (SH6) and sensitive (G935) apple rootstocks under drought and cold stress. A and B, Shoot shriveling phenotypes of tolerant rootstock (R) and sensitive rootstock (S). Scale bars, 45 mm. C-E, Cell membrane permeability (C), relative water content (D), and transcription levels of *MhZAT10* as detected by RT-qPCR under drought stress (E). F-H, Cell membrane permeability (F), relative water content (G), and transcription levels of *MhZAT10* under cold stress (H). Data are means of three replicates ± SD. Statistical significance was determined using Sidak's multiple test: **P*<0.05; ***P*<0.01.

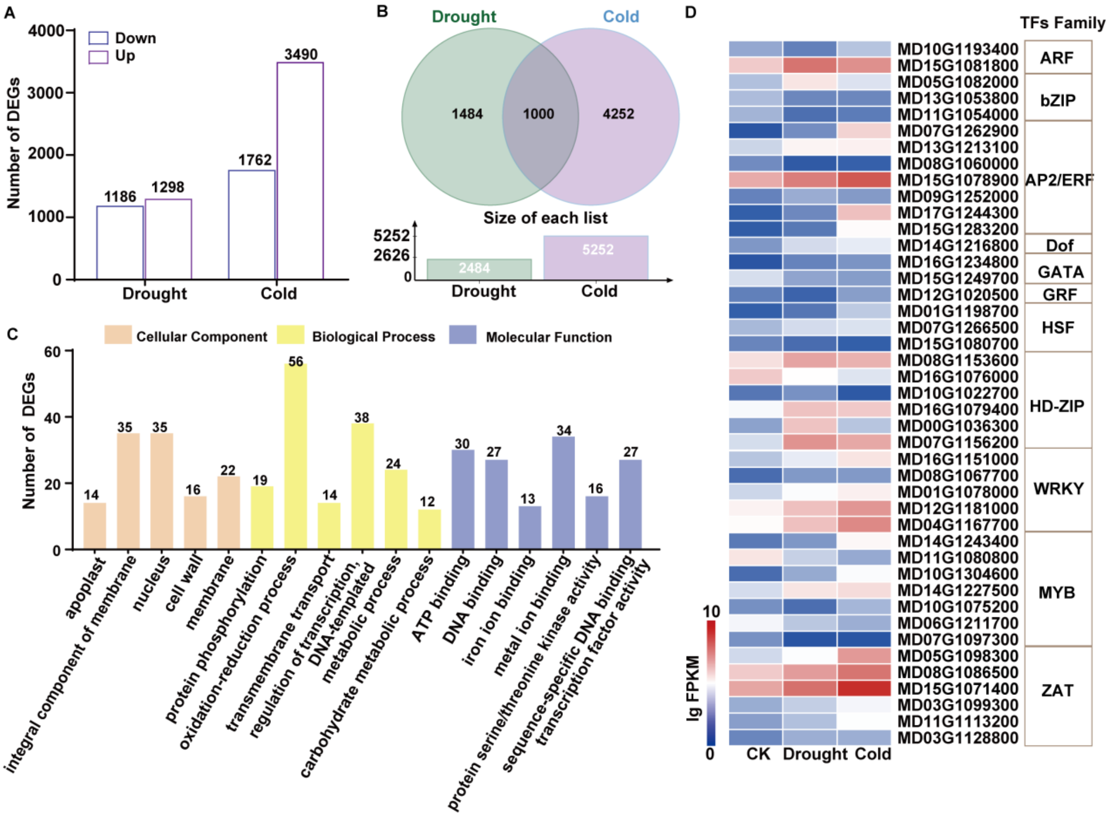

Supplemental Figure S2 RNA-seq analysis of shoot-shriveling tolerant rootstock SH6 under drought and cold stress. A . Differentially expressed genes (DEGs) in response to drought and cold stress. B, Venn diagram analysis of DEGs under drought and cold stress. C, GO enrichment of DEGs responding to both drought and cold stress. D, Expression pattern analysis of transcription factors in response to both drought and cold stress. CK, non-stressed conditions. The color scale represents the values of lg FPKM (FPKM, fragments per kilobase of transcript per million fragments mapped).

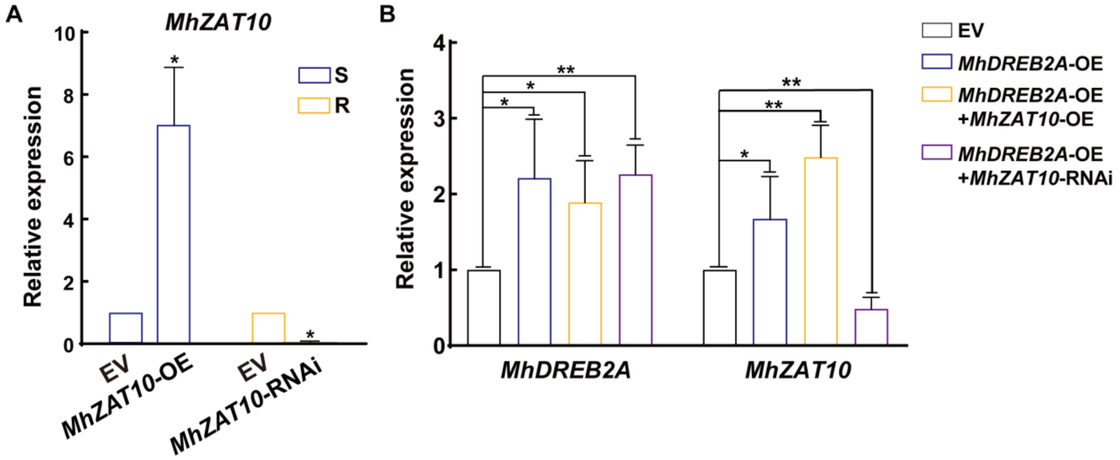

Supplemental Figure S3 Detection of *MhZAT10* and *MhDREB2A* expression levels in transgenic plants. A. Transcription levels of *MhZAT10* as detected by RT-qPCR in transgenic plants. B. Transcription levels of *MhZAT10* and *MhDREB2A* in transgenic plants. S, sensitive rootstock. R, tolerant rootstock. EV, empty vector. OE, overexpression. RNAi, RNA interference. Data are means of three replicates ± SD. Statistical significance was determined using Sidak's multiple test: *P<0.05; **P<0.01.

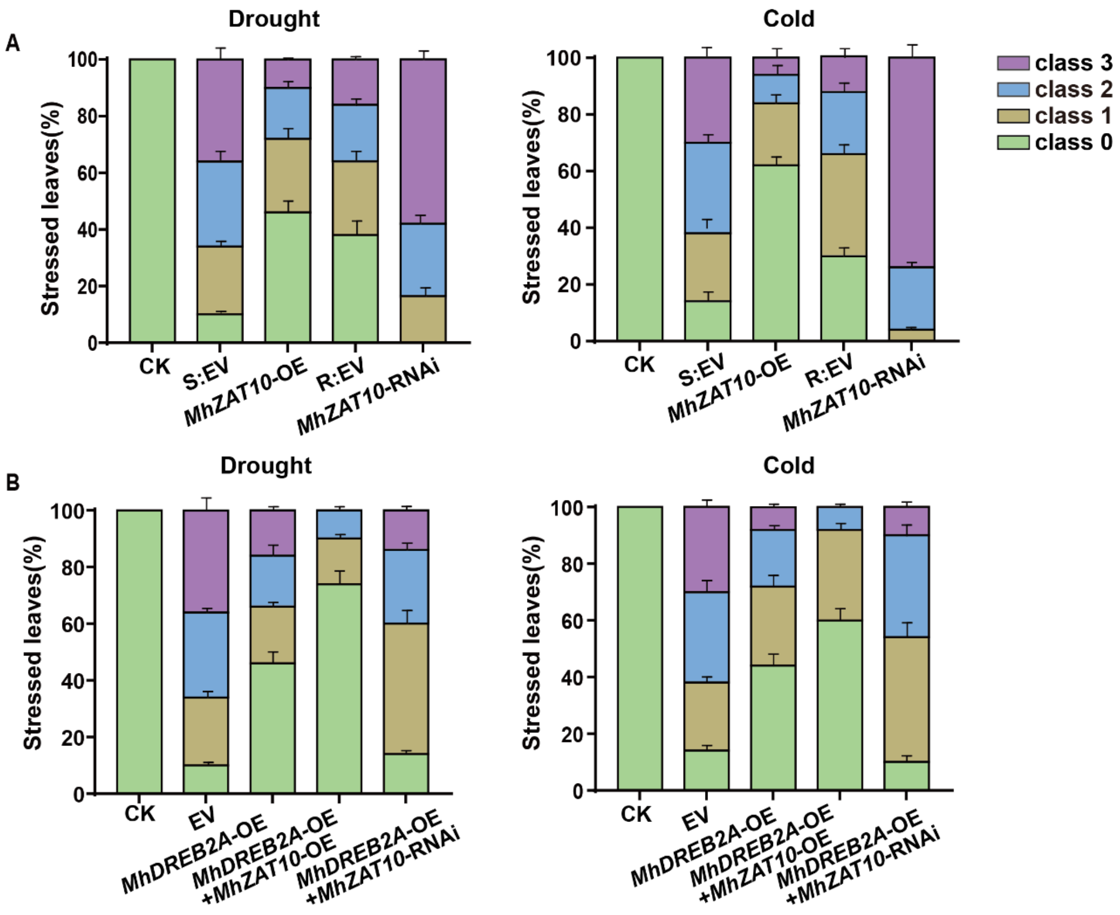

Supplemental Figure S4 Percentage of stressed leaves showing various degrees of severity of stress injury. A, *MhZAT10* transgenic plants on the 7th day of drought and cold stress. B, *MhZAT10* and *MhDREB2A* transgenic plants in the G935 (S) rootstock on the 7th day of drought and cold stress. Class 0, no symptoms; class 1, leaf area with injury < 20%; class 2, 20 to 40% leaf area showing injury; class 3, > 40% leaf area showing injury. S, sensitive rootstock. R, tolerant rootstock. CK, non-transgenic plants. EV, empty vector. OE, overexpression. RNAi, RNA interference. S:EV, transgenic sensitive rootstocks (S) harboring empty vector (EV). R:EV, transgenic tolerant rootstocks (R) harboring empty vector (EV). Data are means of three replicates ± SD, and at least 6 plants were used for each replicate.

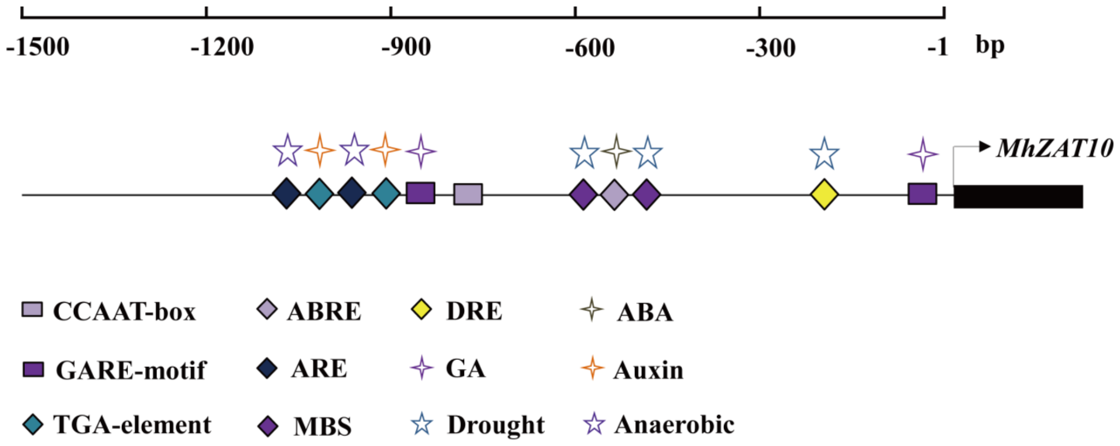

Supplemental Figure S5 *Cis*-elements and putative regulating factors in the *MhZAT10* promoter. These are predicted using PlantCare (http://bioinformatics.psb.ugent.be/webtools/plantcare/html/). ABRE, abscisic acid response element. ARE, anaerobic regulatory element. MBS, MYB binding site. DRE, dehydration response element. ABA, abscisic acid. GA, gibberellic acid.

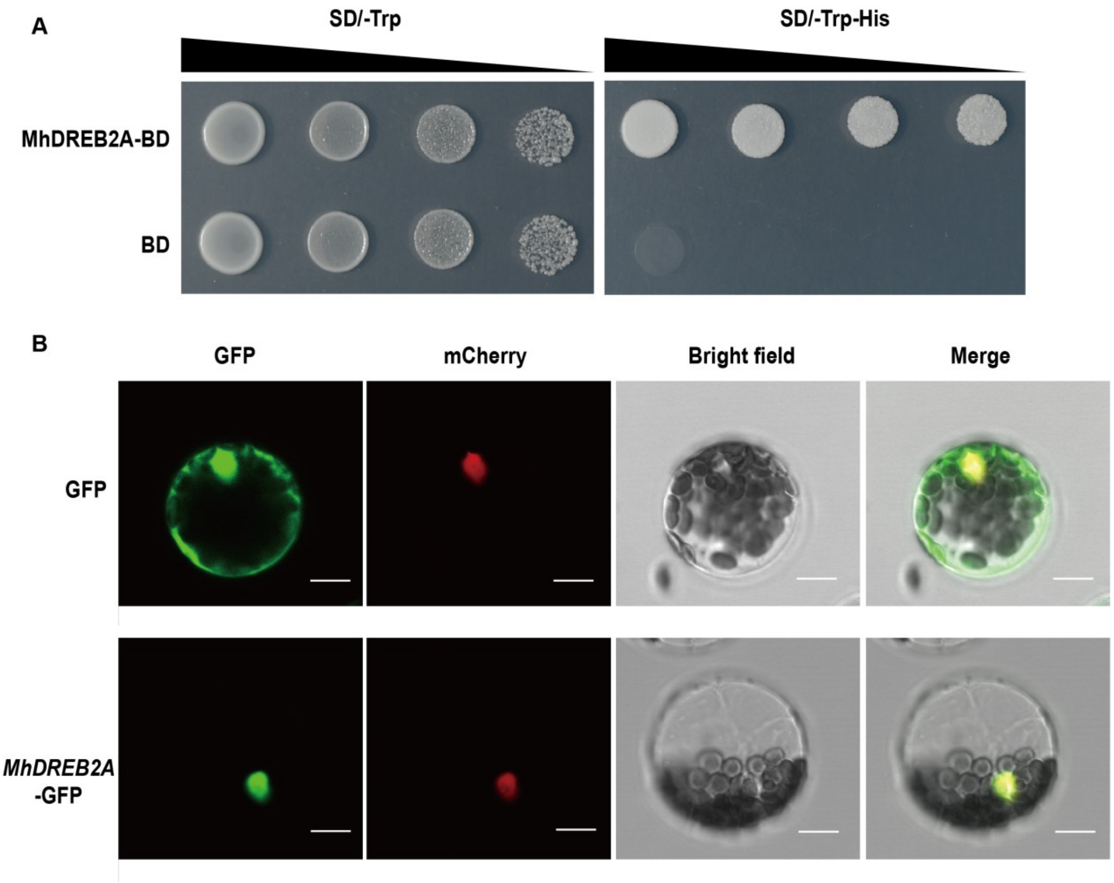

Supplemental Figure S6 Transcriptional activation assay and subcellular localization of MhDREB2A. A, Transcriptional activation assay of MhDREB2A. Each colony was suspended in 10 μL sterile water and then diluted from 10^{-1} to 10^{-4}. B, Subcellular localization of MhDREB2A in Arabidopsis protoplasts. Scale bars, 10 μm.

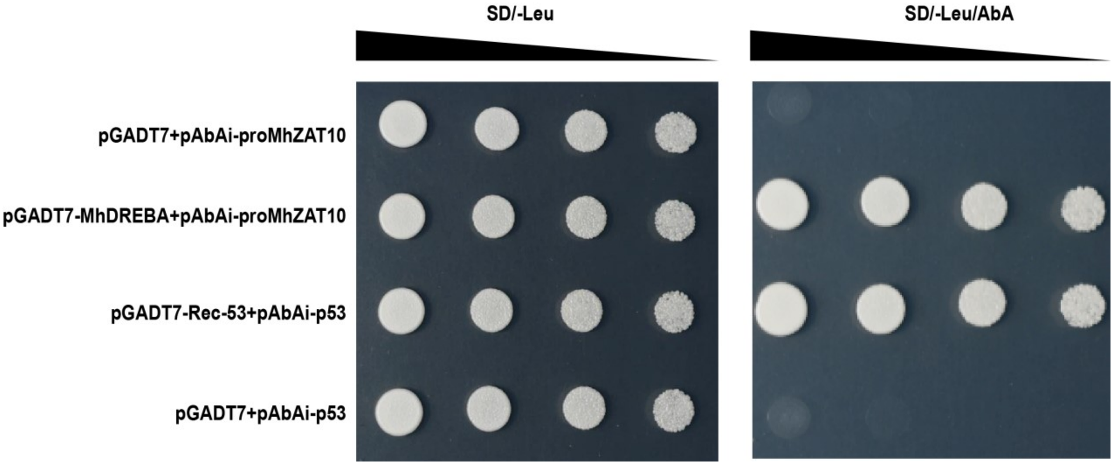

Supplemental Figure S7 Yeast one-hybrid assay showing binding of MhDREB2A to the *MhZAT10* promoter. AbA, Aureobasidin A.

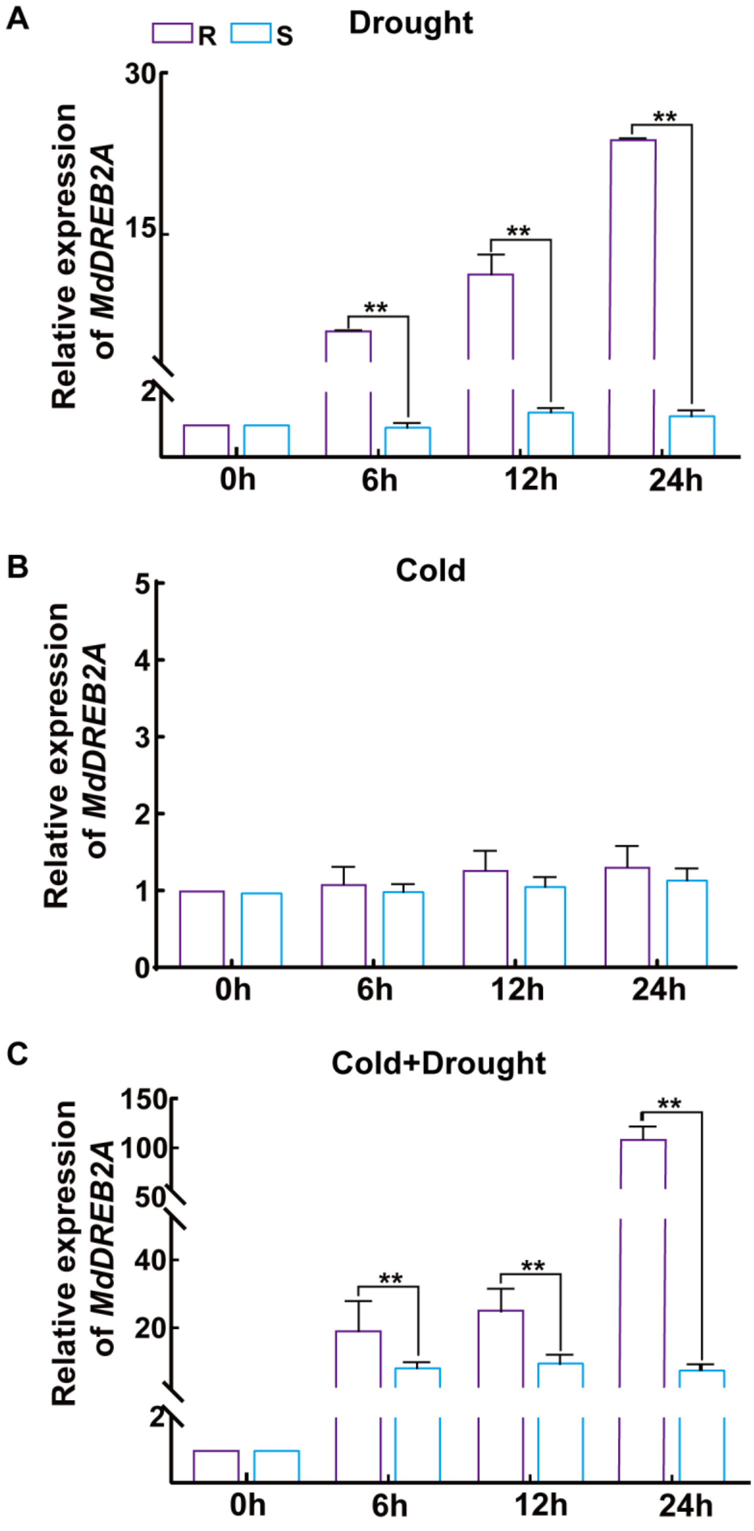

Supplemental Figure S8 Detection of *MdDREB2A* expression levels in shoot-shriveling sensitive rootstock G935 under drought and cold stress. *MdDREB2A* expression levels were detected by RT-qPCR for drought (A), cold (B) and combined stress (C) treatments. Data are means of three replicates ± SD. Statistical significance was determined using Sidak's multiple test: *P<0.05; **P<0.01.

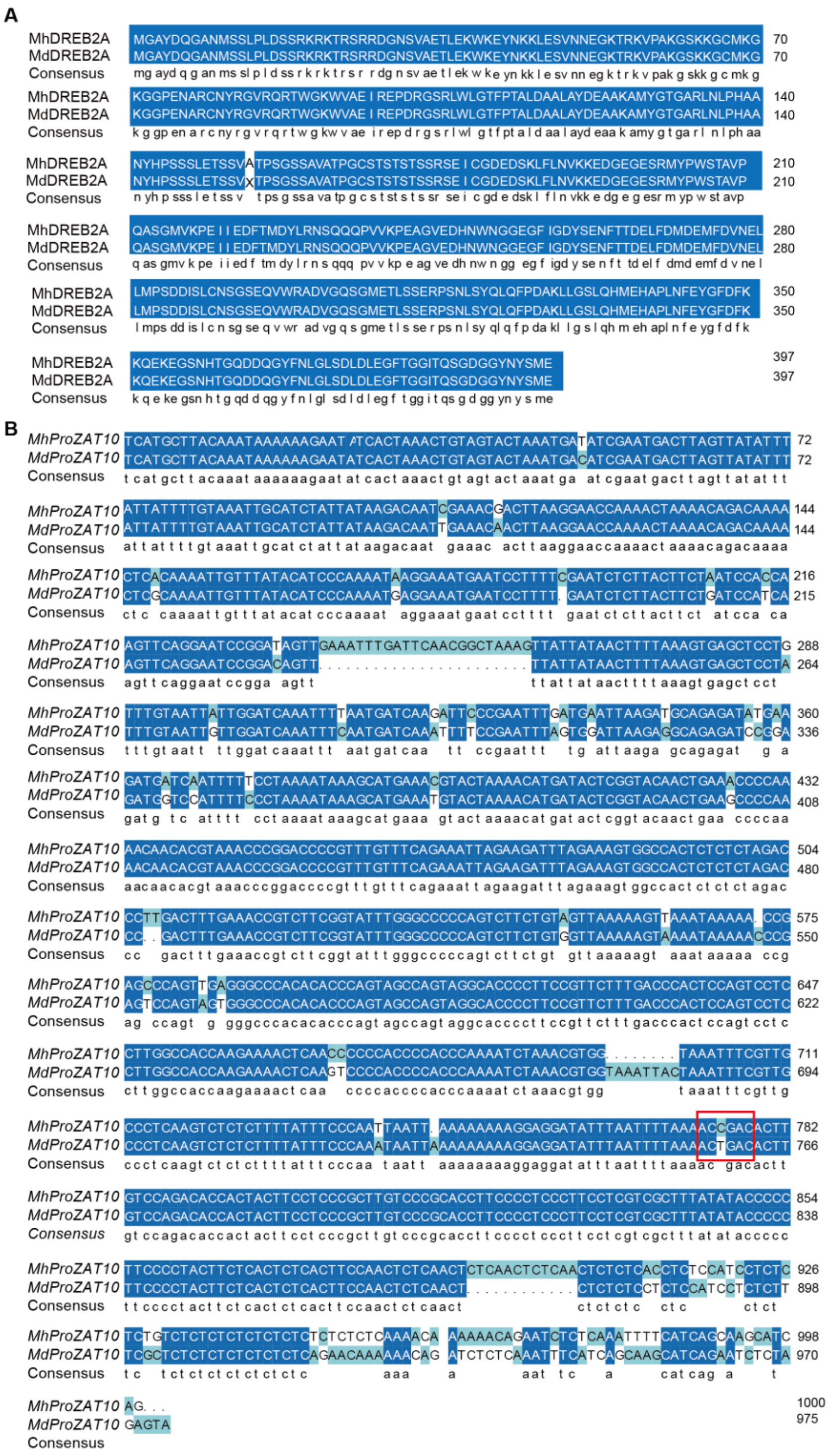

Supplemental Figure S9 Alignment of DREB2A amino acid sequence and *ZAT10* promoter sequence between tolerant (SH6) and sensitive (G935) apple rootstocks. A, Alignment of DREB2A amino acid sequence. B, Alignment of ZAT10 promoter sequence. MhDREB2A and *proMhZAT10* are from SH6 rootstock, MdDREB2A and *proMdZAT10* are from G935 rootstock. Red box indicates the dehydration response element (DRE).

Supplemental Table S1 Primer sequences used for RT-qPCR detection

No.	Genes	Sequence (5′–3′)	GenBank Accession No.
1	*MhZAT10*	F: ACCACAGCCACTAAAGCAACA<br>R: GAGCACTCGTGGGACCTTC	OP562895
2	*MhDREB2A*	F: GAGCAGGCGAGATGGGAACT<br>R: TCCCTTGCCTTTCATACAGC	XM008355947
3	*MhWRKY31*	F: CCGTCCAATGACAACAACCG<br>R: TCCCATCGTCAACCATCGAC	XM008365867.3
4	*MhSnRK2.10*	F: CGGGACTTGAAGTTGGAG<br>R: ACGGTCGATTTTGGTTGTG	KJ868181.1
5	*MhNAC1*	F: CTTCGTCAGCCTCAGATTGT<br>R: CCACTGTATTCTGTATTGGATTG	MF401514.1
6	*MhDREB6.2*	F: TCCCTCTTTCCCTTCTACCTC<br>R: CTGGTGGTGCTTCTCAAATAAC	KX098453.1
7	*MhATG18a*	F: ATGATTCCAGGCTTGCCTGCTTTG<br>R: TGCAGCAAAGTTCCGTCGAGAGTA	KC800804
8	*MhbHLH1*	F: ATGGACGACAGGGAGGAC<br>R: GGAGGAGGAAGAGTCCAC	NM001294038.1
9	*MhMYB88*	F: GCAGCCAGATCTATTCGACGACTCT<br>R: ACAACTTTAAAAGGAGAAGTGAACTCGG	KY569647.1
10	*MhMYB124*	F: ACATTGCAGGCAGCCTGATTTATA<br>R: GTAACTTTAATAGGAGAATTGAACTCTATAC	KY569648.1
11	*MhcyMDH*	F: TCTCGAGGAAGAAGTTGGATGC<br>R: TCAGCTGCTTTTCTCACACATTG	DQ221207.1
12	*MhMYB1*	F: GAATTCGACGATCAAAAAGGCC<br>R: GGATTCGAGCTTGGTGATTAT	KC691248
13	*MhMYB121*	F: TCATCCCCCATCCTCACTACCA<br>R: TCGTTTGCATCTTAGCACTTGC	KC834015.1
14	*MhCIPK6L*	F: GATAAAGAAGAGCGACTCGATGG<br>R: CTTAATCAATCAAGCCGCCGT	NM001328820.1
15	*GUS*	F: TGGGTCAATAATCAGGAAGTG<br>R: CGGGATAGTCTGCCAGTTCA	AF234300.1
16	*β-Actin*	F: CTGAACCCAAAGGCTAATCG<br>R: ACTGGCGTAGAGGGAAAGAA	XM008356922

Supplemental Table S2 Primer sequences used for vector constructions

Purpose	Plasmid Name	Constructs	Sequence (5′–3′)	Enzyme site
Rootstock genetic transformation	pCAMBIA1304	*MhZAT10*-OE	F: CCATGGATGGCTCTGCAAGCTCTCAAC	*NcoI*
			R: GGTAACCCTAATTTTGTTGGTCCTGTGGA	*Bst EII*
	pCAMBIA1304	*MhZAT10*-RNAi	F: GGTAACCATGGCTCTGCAAGCTCTCAAC	*Bst EII*
			R: CCATGGTCCCGAAGGGGTGGCGGTG	*NcoI*
	pCAMBIA1304	*MhDREB2A*-OE	F: CCATGGATGGGAGCTTATGATCAAGG	*NcoI*
			R: GGTAACCTCACATTTCCATCGAATAGTTG	*Bst EII*
Calli genetic transformation	pCAMBIA1304	*ProMhZAT10:GUS*	F: GGATCCCAGTCCTCCTTGGCCACCAAG	*BamHI*
			R: CCATGGCTGATGCTTGCTGATGAAAATTTG	*NcoI*
	pCAMBIA1304	*ProMhWRKY31:GUS*	F: GGATCCGACAACCAATGGGAGATTAAC	*BamHI*
			R: CCATGGAAAATTTAATTAATCTTTTGACGATAC	*NcoI*
	pCAMBIA1304	*ProMhMYB88:GUS*	F: TCTAGAGTCCCCTTTATAAAAGAAAATGC	*XbaI*
			R: CCATGGGGTTTTTAATTTCGCCTCTCTCTCTCAC	*NcoI*
	pCAMBIA1304	*ProMhMYB124:GUS*	F: GGATCCAAACTGAAATATTAAAAACGCAAGC	*BamHI*
			R: CCATGGTTAATTTCACCTCTCTCTCTAGC	*NcoI*
Subcellular localization assay	pEZS-NL-EGFP	MhZAT10-EGFP	F: GAATTCATGGCTCTGCAAGCTCTCAAC	*EcoRI*
			R: GGTACCATTTTGTTGGTCCTGTGGAATC	*KpnI*
		MhDREB2A-EGFP	F: GAATTCATGGGAGCTTATGATCAAGG	*EcoRI*

			R: GGTACCCATTTCCATCGAATAGTTGTAAC	*KpnI*
Transcriptional activation assay	pGBKT7	MhZAT10-BD	F: CATATGATGGCTCTGCAAGCTCTCAAC	*NdeI*
			R: GGATCCCTAATTTTGTTGGTCCTGTGGA	*BamHI*
		MhDREB2A-BD	F: CATATGATGGGAGCTTATGATCAAGG	*NdeI*
			R: GGATCCTCACATTTCCATCGAATAGTTG	*BamHI*
Y1H	pGADT7	MhDREB2A-AD	F: GAATTCATGGGAGCTTATGATCAAGG	*EcoRI*
			R: CTCGAGTCACATTTCCATCGAATAGTTG	*XhoI*
	pGADT7	MhZAT10-AD	F: GAATTCATGGCTCTGCAAGCTCTCAAC	*EcoRI*
			R: CTCGAGCTAATTTTGTTGGTCCTGTGGA	*XhoI*
	pAbAi	ProMhZAT10	F: GGTACCCAGTCCTCCTTGGCCACCAAG	*KpnI*
			R: CTCGAGCTGATGCTTGCTGATGAAAATTTG	*XhoI*
	pAbAi	ProMhWRKY31	F: GGTACCGACAACCAATGGGAGATTAAC	*KpnI*
			R: CTCGAGAAAATTTAATTAATCTTTTGAC	*XhoI*
	pAbAi	ProMhMYB88	F: GGTACCGTCCCCTTTATAAAAGAAAATGC	*KpnI*
			R: CTCGAGTTGGGTTTTTAATTTCGCCTC	*XhoI*
	pAbAi	ProMhMYB124	F: GGTACCAAACTGAAATATTAAAAACGC	*KpnI*
			R: CTCGAGTTGGGTTTTTAATTTCACCTC	*XhoI*
EMSA	pEASY-Blunt E2	MhDREB2A	F: ATGGGAGCTTATGATCAAGG	/
			R: CATTTCCATCGAATAGTTGTAAC	/

	Biotin probe	ProMhZAT10	Biotin-AAAGGAGGATATTTAATTTTAAAACCGACAC TTGTCCAGACACCACTACT	/
	Cold probe	ProMhZAT10	AAAGGAGGATATTTAATTTTAAAACCGACACTTGTC CAGACACCACTACT	/
ChIP-qPCR	pRI101	MhDREB2A	F: GGATCCATGGGAGCTTATGATCAAGG	*BamHI*
			R: ACTAGTCATTTCCATCGAATAGTTGTAAC	Spe*I*
	ProMhZAT10 qPCR detection	P1	F: GAAAACTCAACCCCCCACCCCAC	/
			R: GGAAGGGGGTATATAAAGCGAC	/
		P2	F: GAAAGTGGCCACTCTCTCTAGAC	/
			R: TTGGTGGCCAAGGAGGACTGGAG	/
		P3	F: GGATCAAATTTTAATGATCAAG	/
			R: TAAATCTTCTAATTTCTGAAAC	/
		P4	F: GAACCAAAACTAAAACAGAC	/
			R: AATAATTACAAACAGGAGCTC	/

The letters in red refer to the restriction enzyme sites.

Supplemental Table S3 ZAT family protein list

Organism	Gene ID	Name
Arabidopsis thaliana	AT2G28200	AtZAT5
	AT5G04340	AtZAT6
	AT3G46090	AtZAT7
	AT3G46080	AtZAT8
	AT1G27730	AtZAT10
	AT2G37430	AtZAT11
	AT5G59820	AtZAT12
	AT3G49930	AtZAT13
	AT5G03510	AtZAT14
	AT3G10470	AtZAT15
	AT3G46070	AtZAT16
	AT2G28710	AtZAT17
	AT3G53600	AtZAT18
	AT1G02040	/
	AT2G26940	/
	AT3G19580	/
	AT5G04390	/
	AT5G43170	/
	AT5G67450	/
Malus honanensis	MD02G1211300	MhZAT1
	MD03G1110900	MhZAT2
	MD10G1139600	MhZAT3
	MD04G1063600	MhZAT4
	MD12G1107100	MhZAT5
	MD07G1252200	MhZAT9
	MD08G1086500	MhZAT10
	MD04G1119600	MhZAT11
	MD03G1099300	MhZAT12
